# Core–Shell Metal–Organic Framework Composites: A Review of Synthetic Strategies and Applications in Catalysis and Adsorption

**DOI:** 10.3390/molecules31060956

**Published:** 2026-03-12

**Authors:** Deyun Sun, Shangqing Chen, Haonan Wu, Mingyue Qiu, Youluan Lu, Ningyuan Wang, Qian Ma, Lijuan Shi, Qun Yi

**Affiliations:** Key Laboratory of Green Chemical Engineering Process of Ministry of Education, School of Chemical Engineering and Pharmacy, Wuhan Institute of Technology, Wuhan 430205, China; 12401010018@stu.wit.edu.cn (D.S.); sqchen@wit.edu.cn (S.C.); 22401010050@stu.wit.edu.cn (H.W.); 12501010001@stu.wit.edu.cn (M.Q.); 12201010003@stu.wit.edu.cn (Y.L.); 12501010016@stu.wit.edu.cn (N.W.); 24079304@wit.edu.cn (Q.M.)

**Keywords:** metal–organic frameworks, core–shell MOFs, composite materials, synthetic strategies, multidomain applications

## Abstract

Core–shell metal–organic framework (MOF) composites, owing to their unique structural advantages, have emerged as a prominent research focus in the field of chemistry, advanced materials and chemical engineering. By integrating MOFs with other functional components such as MOFs, covalent organic frameworks (COFs), metal oxides, carbon materials, ionic liquids or polymers into synergistic heterogeneous architectures, coreshell MOFs can markedly enhance physicochemical stability and enable diversified functional performances. This work provides a systematic overview of the major construction strategies for these materials, including in situ growth, self-templating, seed-mediated methods, one-pot synthesis and post-synthetic modification. It also summarizes recent applications in catalysis (thermal, electrocatalytic and photocatalytic processes) as well as gas adsorption and separation (such as CO_2_ capture from flue gas, natural gas purification and acetylene separation). The final section discusses future research directions, including a deeper understanding of interfacial growth mechanisms, the development of green and scalable synthesis routes, the validation of engineering-oriented applications, and the integration of machine learning with high-throughput computation for structural prediction and accelerated materials screening, thereby providing important guidance for the development of high-performance core–shell MOFs.

## 1. Introduction

Metal–organic frameworks (MOFs) were first synthesized by the Yaghi group in 1995 [1]. They are unique crystalline porous materials composed of metal ions or metal clusters as structural nodes [2,3,4,5]. Due to the diversity of metal centers and organic connectors, MOFs exhibit adjustable structures and properties through precise design at the molecular level [6,7]. In addition, their pore sizes are adjustable, with controllable topologies and diverse functionalization pathways [8,9,10]. Compared with traditional inorganic porous materials, such as molecular sieves, zeolites, activated carbon, etc., MOFs have greater design flexibility and structural tunability [11,12]. The pore size, surface polarity and chemical reactivity may be modified selectively by altering ligand length, functional groups or metallic center type [13,14].

It has been shown that MOFs have broad application prospects for gas adsorption and separation [15,16,17], catalysis [18,19], and energy storage and conversion [20,21]. Yet, despite great progress with MOFs, they still have a number of issues that matter. Some MOF structures have low chemical stability and may collapse structurally or lose performance when subjected to humidity, acid or base, and high temperature conditions [22,23]. It has been reported that especially MOFs containing flexible coordination bonds with metal ions Zn^2+^ or Cu^2+^ are liable to undergo hydrolysis in humid or acidic gaseous conditions, thereby leading to a considerable loss of framework stability and functionality [24,25,26]. Moreover, owing to the poor pore size matching between the MOF materials and gas molecules, and their somewhat single surface chemical environment, the selectivity of most MOFs for gas separation is poor [27,28]. Another issue is that most conventional MOFs are electrical insulators, which greatly limits their use in electrochemical catalysis, sensors, and electronic devices [29,30].

In order to solve these problems, researchers have proposed a wide range of improvement strategies. These strategies include introducing more stable metal nodes (Zr^4+^, Ti^4+^) [31,32,33], developing metal-free covalent organic frameworks (COFs) [34,35], and the incorporation of conductive materials (carbon materials and metal oxides) [36,37], and the construction of hierarchical pore structures [38,39]. Core–shell MOF composites have attracted considerable attention owing to their pronounced structural and functional synergies. In these composite structures, a MOF or another type of functional material serves as the central “core” component, which is responsible for providing the primary structural integrity or catalytic activity. Surrounding this core, a “shell” possessing tailored physical and chemical properties is applied to the surface, resulting in the formation of a hierarchical composite material. The interaction between the core and the shell is maintained through various bonding mechanisms, which may include chemical bonds, electrostatic interactions, hydrogen bonding, or van der Waals forces, ensuring a stable integration of both components [40,41]. The shell layer design not only greatly improves the physicochemical stability of the MOF but also allows for interfacial regulation and environmental control. To illustrate, one can utilize hydrophobic shells for enhanced moisture stability [42], employ conductive shells for better electron conductivity [43], and apply porous shells for improved molecular sieving and reaction selectivity [44]. The benefits of this approach include nanoscale functional componentization, improved dispersibility, enhanced cycling stability, and the induction of new electronic structures or catalytic activity at the core–shell interface. Specifically, in terms of CO_2_ capture and conversion, core–shell MOF composites have the potential to merge the high adsorptive capacity of MOFs with the catalytic functionalities of ionic liquids (ILs) or metal oxides, which could enhance the overall efficiency significantly and open new design avenues for practical applications [45,46,47].

Recently, several review articles have examined core–shell MOFs and related organic framework materials in terms of their photocatalysis [48,49] and electrochemical applications [50]. These reviews essentially summarize the common synthesis methods and structural regulation strategies, and their recent developments in applications towards environmental remediation and energy conversion. Nonetheless, a majority of these studies are limited to a single application field or a particular structural type, and the overall classification of core–shell MOFs, the intrinsic relationships among different construction strategies and their multidisciplinary applications are still lacking. In light of this, the present review aims to give a systematic account of the types of core–shell MOFs, their syntheses and recent progress in research. We first introduce the different types of core–shell MOFs, followed by an in-depth discussion of the major construction strategies and related technical methods. Furthermore, we highlight representative applications of core–shell MOFs in gas adsorption and catalysis, and other fields, and outline potential directions for future development. Through a comprehensive examination and analysis of the current literature, this review seeks to furnish valuable insights for researchers in the field and to stimulate further innovation and expansion of core–shell MOF materials.

## 2. Types of Core–Shell MOFs

The classification of core–shell MOF composites is typically based on the types of constituent materials. According to material type, core–shell MOFs mainly include the following categories: (1) single MOF core/shell structures (MOF@MOF), in which both the core and shell are composed of the same or different types of MOFs, achieving functional complementarity or pore structure optimization through interfacial engineering [51,52,53]. (2) MOF@COF, where COFs are used as the shell to enhance structural stability or regulate molecular sieving performance [54,55,56]. (3) MOF@oxide materials are constructed by coating MOFs with inorganic oxide layers. This strategy enhances the mechanical strength and thermal stability of the materials, while preserving the intrinsic porous structure and active sites of the MOF cores [57,58]. (4) MOF@carbon materials are obtained by coating MOFs with carbon-based shells. In addition to improving structural stability, the carbon shell imparts electrical conductivity and can enhance catalytic performance [36]. (5) MOF@ionic liquid/polymer, where functional ILs or polymers as the shell can improve gas adsorption performance, enhance separation selectivity and catalytic activity [59,60,61,62]. (6) A@MOF (A represents carbon materials, oxides, or polymers) consists of a core made from either inorganic or organic materials, while MOFs function as the outer shell, integrating molecular sieving, catalytic, or carrier roles [63,64,65]. This classification approach offers precise direction for the focused design and real-world application of core–shell MOFs.

### 2.1. MOF@MOF Composites

MOF@MOF composites refer to a class of heterostructures constructed through interfacial engineering, formed from either similar or different MOFs, in which one MOF serves as the core while the other forms a continuous shell [66]. The core MOF provides a high surface area and a stable porous framework, facilitating substrate adsorption and molecular diffusion, while the shell MOF can modulate pore size, enhance selectivity, and introduce additional active sites, thereby improving catalytic performance and separation efficiency. MOF@MOF composites can be prepared using various strategies, including epitaxial growth, seed-mediated growth, one-pot synthesis, surfactant-assisted growth, and ligand/metal ion exchange, allowing precise control over the structure and functionality under tailored nucleation and growth conditions. These core–shell architectures exhibit excellent performance in gas separation, catalysis, and energy storage, and can be rationally designed to meet the requirements of different reaction systems, providing an effective strategy for the development of high-performance functional MOF materials [67].

#### 2.1.1. Epitaxial Growth

**Figure 1:**
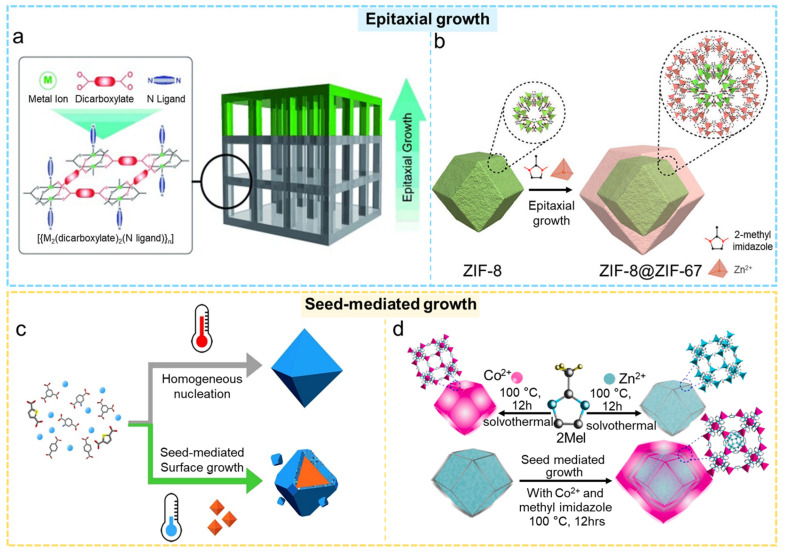
(**a**) Synthetic scheme of preparation of core–shell ZIF-8@ZIF-67 and ZIF-67@ZIF-8 [69]; (**b**) schematic illustration of the synthesis process of ZIF-8@ZIF-67 [72]; (**c**) seed-mediated, low-energy strategy for the growth of core–shell MOFs [73]; (**d**) schematic diagram of the synthesis process of ZIF-8, ZIF-67 and core–shell structure ZIF-8@ZIF-67 [74].

Epitaxial growth is one of the most common methods to construct core–shell MOF@MOF composites. The approach commences with the preparation of the MOF core particles, followed by the induced, oriented growth of a second MOF on the core’s surface. This results in the shell layer being able to uniformly coat the MOF core with a highly matched lattice orientation [67,68]. The epitaxial growth process of the core and shell is related to lattice compatibility, surface chemical groups, and the regulation of the solvent environment. By tuning parameters such as reactant concentration, pH, solvent type, and temperature, precise control over shell thickness, morphology, and interfacial structure can be achieved. Compared with other assembly strategies, epitaxially grown core–shell MOF@MOF structures exhibit superior interfacial stability and higher structural continuity. As early as 2009, Furukawa and colleagues first employed an epitaxial growth strategy to synthesize core–shell MOF@MOF composites ({Cu_2_(ndc)_2_-(dabco)}_n_@{Zn_2_(ndc)_2_-(dabco)}_n_) [69]. The structural relationship between the core crystal and the shell crystal was clarified using surface X-ray diffraction (XRD), thereby laying the groundwork for developing core–shell MOF@MOF materials through solution-based epitaxial growth (Figure 1a). In later studies, this research team broadened this methodology and effectively created a range of core–shell MOF@MOF materials, along with MOF@MOF@MOF composites featuring sandwich-like structures [70,71].

In 2018, a core–shell composite, ZIF-8@ZIF-67, was successfully developed by Li et al. through an epitaxial growth approach, where ZIF-8 acted as the core and ZIF-67 as the outer shell (Figure 1b) [72]. Using this material as a precursor, they fabricated a mixed nanocomposite (CoP/NCNHP) through a pyrolysis–oxidation–phosphorization process. Transmission electron microscopy (TEM) clearly revealed the core–shell structure, demonstrating the synergistic effect between highly active CoP nanoparticles and NCNHP, resulting in excellent electrocatalytic performance. Sepehrmansourie et al. also employed an epitaxial growth approach to grow UiO-66 on the surface of NH_2_-MIL-125, thereby producing a core–shell MIL-125@UiO-66 composite [51]. They subsequently modified MIL-125@UiO-66 with g-C_3_N_4_, and for the first time constructed a dual Z-scheme MIL-125@UiO-66/g-C_3_N_4_ heterostructure. Spectroscopic investigations validated the epitaxial development of UiO-66 on NH_2_-MIL-125 and the effective modification of g-C_3_N_4_ on the surface of UiO-66@MIL-125. This core–shell MOF@MOF composite displayed outstanding performance in photodegradation.

#### 2.1.2. Seed-Mediated Growth

Compared with epitaxial growth, the seed-mediated growth method offers greater controllability and broader applicability. This method utilizes pre-synthesized MOF crystals as seed materials, where the active sites, functional groups, or residual ligands present on their surfaces facilitate the nucleation and subsequent development of a second MOF on the outer layer [75,76]. Unlike epitaxial growth—which requires a high degree of lattice matching—seed-mediated growth is less dependent on lattice compatibility between the core and shell, making it more suitable for constructing core–shell MOF@MOF systems with large lattice parameter differences or distinct ligand frameworks. By modifying factors like the concentration of shell MOF precursors, polarity of the solvent, pH levels, and the speed at which precursors are added, one can attain precise regulation of shell thickness, morphology, and uniformity [75]. Furthermore, introducing functional groups (e.g., -NH_2_, -COOH, ionic ligands) onto the seed surface can further enhance nucleation selectivity and suppress the formation of undesired phases. Owing to its simplicity and versatility, the seed-mediated growth technique has seen extensive application in the creation of different core–shell MOF@MOF structures [77].

Jiang et al. developed a low-energy, seed-mediated growth strategy for constructing core–shell MOF@MOF architectures [73]. By directly introducing pre-synthesized MOF seeds into the reaction system, they achieved rapid growth of the shell MOF under mild conditions of only 50 °C, with growth rates tens of times faster than those of conventional low-temperature solvothermal methods. The core–shell materials produced maintained the high crystallinity, ample porosity, and high stability of the seed crystals, while also displaying significantly improved catalytic performance. In the aromatic iodination of 2-methoxynaphthalene, the strong Lewis acidity emerging in the seed-mediated MOFs rendered their activity an order of magnitude higher than that of the seed MOFs themselves. Moreover, Panda et al. constructed a ZIF-8@ZIF-67 core–shell structure using a seed-mediated growth approach [74]. The successful creation of this heterogeneous core–shell structure was verified through high-resolution TEM, HAADF-STEM (high-angle annular dark field scanning transmission electron microscopy), and elemental mapping analyses. The heterointerface can significantly enhance the triboelectric performance and show good stability and energy conversion efficiency in current-carrying triboelectric nanogenerators (TENGs). Dai et al. utilized a seed-mediated growth strategy to produce a core–shell composite of NH_2_-MIL-125@ZIF-67 with MIL-125 as the core and ZIF-67 as the shell [77]. The conjoined interaction taking place between the core and the shell vastly improved the adsorption ability for metal ions which shows excellent promise for the removal of Cd(II) contamination from water.

#### 2.1.3. One-Pot Synthesis

The one-pot synthesis of core–shell MOF@MOF composites has garnered considerable interest due to its simplicity, high process continuity, and ease of scaling up. The strategy enables a continuous “core-first, shell-later” growth mechanism within a single reaction system, in which the metal ions and organic linkers of different MOFs are added either simultaneously or sequentially under identical solvent and temperature conditions [78,79]. The nucleation rates and growth rates of the two MOFs are different. The MOF that has the faster nucleation rate will form the core first. Subsequently, the MOF that has the slower nucleation rate and lower reactivity with ligand will spontaneously grow on top, forming a thicker coating on the core to form the shell. By adjusting the experimental parameters including the metal–ligand ratio, the addition sequence of the ligands and the temperature of the reaction, precise control over the shell thickness, interfacial structure and compositional gradients can be achieved.

**Figure 2:**
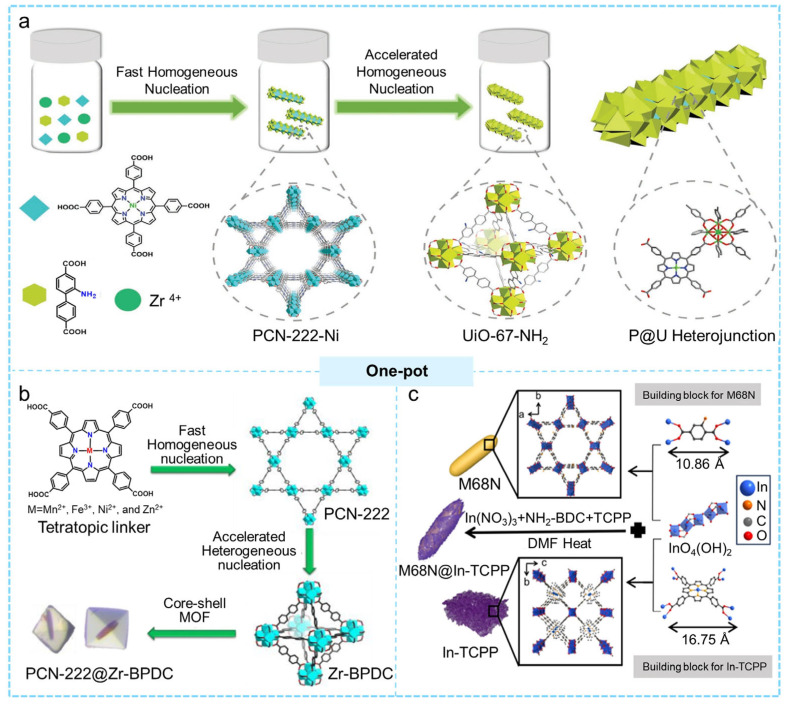
(**a**) One-pot synthesis of PCN-222-Ni@UiO-67-NH_2_ (P@U) heterojunction [80]; (**b**) kinetically controlled synthesis of hybrid core–shell PCN-222@Zr-BPDC [81]; (**c**) block size and crystal structure of M68N and In-TCPP are illustrated, and the construction process of M68N, In-TCPP and M68N@In-TCPP core–shell MOFs structures prepared by one-pot method is illustrated [83].

The one-pot approach effectively minimizes material loss while improving the integrity and reproducibility of the core–shell materials, making them widely applicable in catalytic reactions and pollutant elimination. The authors Cao et al. in 2022 utilized the difference in nucleation kinetics between two MOF materials to create a multilevel core–shell MOF@MOF (PCN-222-Ni@UiO-67-NH_2_ (P@U)) architecture using a straightforward one-pot approach (Figure 2a) [80]. Moreover, the resulting heterostructure inherited the parent MOFs’ high porosity, crystallinity, and robustness, but increased significantly the photogenerated charge separation, allowing for the overall photoreduction of CO_2_. Remarkably, without the use of any sacrificial agent, it produced a record-high yield of HCOOH. Zhou et al. employed a one-pot strategy to synthesize hybrid core–shell MOFs featuring lattice mismatch (e.g., PCN-222@Zr-BPDC) guided by nucleation kinetics (Figure 2b) [81]. This core–shell material displayed strong catalytic activity in olefin epoxidation. To further validate the broad applicability of this kinetics-directed approach, the authors additionally prepared three other lattice-mismatched core–shell MOFs (PCN-134@Zr-BTB, PCN-222@Nu-1000, and La-TCPP@La-BPDC). Hu et al. prepared a core–shell MOF(Ce)@MOF(Zr) using a one-pot method, which exhibited rapid fluoride capture in both acidic and alkaline solutions [82]. Zhu and colleagues developed the core–shell MOF M68N@In-TCPP using a straightforward one-pot method that capitalizes on the competitive nucleation and growth of two organic linkers that are coordinated to indium (In) nodes (Figure 2c) [83]. The shell of In-TCPP surrounds the core of In-NH_2_-MIL-68, resulting in a robust heterostructure. The In-TCPP shell significantly boosts the adsorption of CO_2_ and the absorption of visible light, whereas the In-O sites located in the M68N core effectively catalyze the oxidation of H_2_O, collectively facilitating the production of substantial amounts of formic acid (HCOOH) and hydrogen peroxide (H_2_O_2_).

#### 2.1.4. Surfactant-Assistant Growth

In the synthesis of nanomaterials, surfactants are widely employed to regulate morphology, particle size, crystal facet orientation, porosity, and surface properties. Additionally, they can improve the interactions between the substrate and the deposited materials, which makes them important for the development of hybrid structures [84,85,86]. In the process of creating core–shell MOF@MOF composites, the guest MOF present in the solution frequently experiences homogeneous nucleation and growth. This leads to a combination of both host and guest MOFs instead of a clearly defined core–shell structure. Surfactants can preferentially adsorb onto the surface of the host MOF, increasing interfacial affinity and directing the deposition of guest-metal ions. This promotes heterogeneous nucleation and uniform shell growth, ultimately enabling the successful formation of core–shell MOF@MOF structures [87,88]. Compared with traditional epitaxial growth, the surfactant-assisted strategy is simpler and more versatile, accommodating various metal ions and surfactants. However, it still has limitations: surfactants can become unstable when exposed to high temperatures, and guest MOFs frequently bond to the host surface in a random manner, complicating the process of achieving site-specific growth.

**Figure 3:**
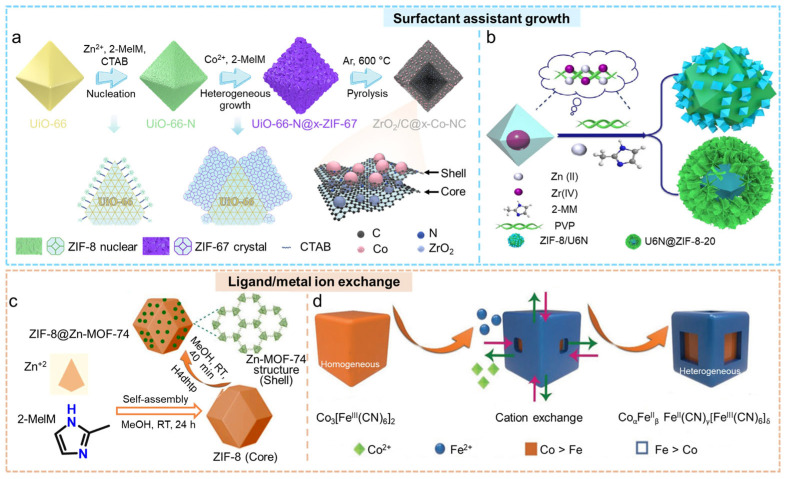
(**a**) Schematic diagram of the preparation process of ZrO_2_/C@x-Co-NC [89]; (**b**) preparation of U6N@ZIF-8 and ZIF-8/U6N [91]; (**c**) synthesis of ZIF-8 and ZIF-8@Zn-MOF-74 composite nanocrystals [92]; (**d**) preparation reaction diagram of core–shell Prussian blue analogs (PBAs), Fe^2+^ ions (pink arrows) replace Co^2+^ ions (green arrows) [93].

Shen et al. facilitated the development of ZIF-8 and ZIF-67 nuclei on the surface of UiO-66 with the help of the surfactant cetyltrimethylammonium bromide (CTAB) (Figure 3a) [89]. Through additional pyrolysis, the researchers produced highly mesoporous derivatives that contain core–shell arrangements of ZrO_2_ and Co nanoparticles. Thanks to the beneficial electronic properties and effective mass transport of reactants and intermediates facilitated by the reductive Co sites and Lewis-basic ZrO_2_, the resultant core–shell catalyst achieved an impressive yield (99.5%) of *N*-(4-methoxyphenyl)formamide from the interaction of CO_2_ with nitroarenes. Stylianou et al. first immersed the core MOF (UiO-67) in a solution containing the precursor of the shell MOF along with a structure-directing surfactant (CTAB), followed by solvothermal treatment [90]. This process facilitated the stepwise growth of the shell around the core, enabling a Lego bricks-like hierarchical assembly in which multilayer shells could be constructed in multiple directions. The resulting core–shell structures significantly enhanced the quantum yield. Xiong et al. developed a core–shell structure composed of MOF@MOF (U6N@ZIF-8-20), utilizing UiO-66-NH_2_ as the core and ZIF-8 as the shell, while employing polyvinylpyrrolidone (PVP) as a surfactant [91]. The capping effect produced by PVP altered the growth characteristics of ZIF-8, changing it from its usual dodecahedral shape to a sheet-like configuration, resulting in a final core–shell material with a three-dimensional morphology resembling an “agarose-like” morphology (Figure 3b). In comparison to UiO-66-NH_2_ and ZIF-8, the U6N@ZIF-8-20 demonstrates enhanced thermal and water stability. This material is characterized by its functional groups, including amino, hydroxyl, and carboxyl moieties, which possess a strong affinity for rare-earth elements. Additionally, it features a significant specific surface area, high porosity, a distinctive 3D agarose-like configuration, and a multitude of accessible sites for adsorption. As a result, it showcases exceptional performance in adsorbing Er(III) and exhibits swift and efficient kinetics for adsorption.

#### 2.1.5. Ligand/Metal Ion Exchange

In MOF construction, a single metal ion can coordinate with multiple organic linkers to form stable structures [14,94]. This intrinsic flexibility endows MOFs with diverse structural tunability and provides a foundation for the subsequent structural control of core–shell MOF@MOF architectures. The formation of core–shell MOF@MOF materials can be achieved through two exchange processes that are mechanistically similar yet fundamentally distinct in their mode of action. The first is ligand exchange, which relies on the ability of metal nodes in MOFs to coordinate with different organic linkers. When new linkers with higher affinity for the metal centers are introduced, they preferentially substitute the original ligands at the outer surface of the host MOF. This gradual replacement induces the formation of a second framework at the interface. As this substitution occurs from the outside to the inside, its progression is dictated by reaction kinetics, which can therefore be controlled by modifying factors like the concentration, temperature, and duration of the reaction [95]. Sadatnia et al. utilized ZIF-8 as the host MOF and incorporated 2,5-dihydroxyterephthalic acid (H_4_dhtp) through a post-synthetic ligand exchange method to create a hierarchical core–shell MOF, ZIF-8@Zn-MOF-74, which included open metal sites (OMS) (Figure 3c) [92]. The ZIF-8@Zn-MOF-74 exhibited significant adsorption capacities for CO_2_, C_2_H_4_, and C_3_H_6_. Ma et al. employed Ni-BDC as the precursor MOF and incorporated tetrakis(4-carboxyphenyl)porphyrin (TCPP) through ligand exchange to synthesize a Ni-BDC@Ni-TCPP composite with a nanosheet-like architecture [96]. The presence of numerous exposed catalytically active sites improved electron transfer, whereas the nanosheet structure promoted electron diffusion over the surface of the catalyst. Consequently, Ni-BDC@Ni-TCPP-3 demonstrated outstanding catalytic performance, reaching a hydrogen evolution rate of 428.0 μmol·g^−1^. In contrast, metal ion exchange typically occurs after the framework has been stabilized or has undergone ligand exchange. By exploiting the differences in coordination affinity between various metal ions and the same organic linker, externally introduced metal ions can be selectively incorporated into the existing network without disrupting the overall framework [97]. This process enables the redistribution or stratification of metal centers within the material. For example, cation exchange between Fe^2+^ and Co^2+^ can generate Fe-Co heterostructures with nonuniform metal compositions and open cage architectures (Figure 3d) [93]. Overall, ligand exchange primarily governs the evolution of the organic linker environment and interfacial structure, whereas metal ion exchange imparts new functional sites by altering the metal nodes themselves.

### 2.2. MOF@COF Composites

In composite materials consisting of MOF@COF, MOFs usually function as the nuclei or substrates for the growth of a COF, providing interfacial support that guides the oriented nucleation and construction of COFs on their outer surfaces [98]. Owing to the structural stability of MOFs and the abundance of accessible coordination sites on their surfaces, MOF@COF composites exhibit exceptional structural integrity and strong interfacial adhesion, making them highly attractive for researchers [99]. Various synthetic strategies have been developed to promote COF growth, including the one-pot reaction, in situ growth, and amorphous-to-crystalline transformation. Although these methods differ in procedure, they share a common goal: to achieve continuous, uniform, and well-controlled COF growth on the MOF surface, thereby yielding more stable core–shell MOF@COF materials.

#### 2.2.1. One-Pot Reaction

**Figure 4:**
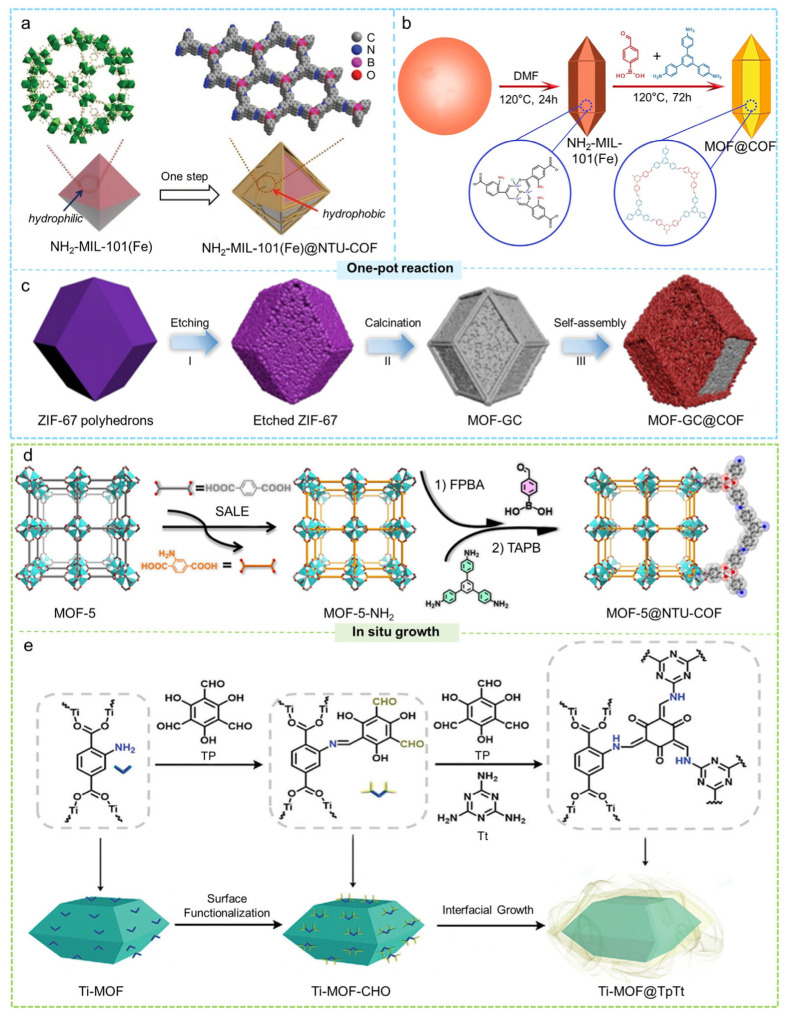
(**a**) One-pot synthesis process diagram of NH_2_-MIL-101(Fe)@NTU-COF [101]; (**b**) schematic illustration of the preparation of core–shell MOF@COF composite [102]; (**c**) schematic representation of the formation process of MOF-GC@COF heterostructure [104]; (**d**) schematic diagram of the construction and preparation of MOF-5@NTU-COF core–shell composites [106]; (**e**) construction and synthesis schematic diagram of Pd modified Ti-MOF@TpTt composite structure [107].

The one-pot reaction uses a pre-synthesized MOF as the core and induces the formation of a COF shell within a single reaction step, greatly simplifying the synthetic process. Nonetheless, this method demands careful regulation of reaction variables, such as the solvent, temperature, and linker concentration, to guarantee that the COF preferentially nucleates on the surface of the MOF rather than in the bulk solution [99,100]. In 2019, Cai et al. employed mesoporous NH_2_-MIL-101(Fe) as a scaffold and introduced two monomers conducive to COF formation, namely, 4-formylphenylboronic acid (FPBA) and 1,3,5-tris(4-aminophenyl)benzene (TAPB) [101]. A condensation reaction took place between the amino groups present in NH_2_-MIL-101(Fe) and FPBA, which anchored FPBA to the surface of the MOF (Figure 4a). The remaining -B(OH)_2_ groups on FPBA acted as nucleation sites, facilitating the further development of NTU-COF. This process resulted in the successful synthesis of a core–shell composite, NH_2_-MIL-101(Fe)@NTU-COF, via a one-pot reaction. The hydrophobic NTU-COF shell that enveloped the NH_2_-MIL-101(Fe) improved the pore characteristics and adjusted the balance between hydrophilicity and hydrophobicity of the material, thereby allowing NH_2_-MIL-101(Fe)@NTU-COF to demonstrate outstanding conversion rates and selectivity in the oxidation process of styrene. Similarly, Ma et al. synthesized a hydrophobic and stable core–shell MOF@COF composite (NH_2_-MIL-101(Fe)@TAPB-FPBA-COF) via a one-pot Schiff base reaction [102]. NH_2_-MIL-101(Fe), featuring a hydrophilic surface, high specific surface area, and amino functional groups, served as the MOF core, while TAPB-FPBA-COF, synthesized from TAPB and FPBA, provided a chemically stable and hydrophobic shell. The hydrophobic “shielding” effect of the shell significantly enhanced the hydrophobicity and stability of the core–shell material (Figure 4b). Furthermore, this one-pot strategy has been successfully applied to develop different kinds of core–shell MOF@COF composites. For instance, He and colleagues utilized NH_2_-MIL-125(Ti) as the core component and adopted the exceptionally stable TTB-TTA system, specifically 4,4′,4″-(1,3,5-triazine-2,4,6-triyl)tribenzaldehyde (TTB) along with 4,4′,4″-(1,3,5-triazine-2,4,6-triyl)trianiline (TTA), to serve as the precursor for the shell, facilitating the preparation of the core–shell composite NH_2_-MIL-125(Ti)@TTB-TTA. The resulting material showed strong photocatalytic degradation activity toward methyl orange [103]. Zhang et al. reported a new core–shell heterostructure in which ZIF-67, etched with a weak organic acid, was thermally converted into MOF-GC [104]. Using a one-pot reaction, they successfully assembled a COF layer constructed from 1,3,5-triformylphloroglucinol (TFP) and 2,5-dimethyl-p-phenylenediamine (DMPA) onto the MOF-GC surface, yielding the core–shell MOF-GC@COF material. This composite exhibited outstanding formaldehyde adsorption performance (Figure 4c). Zheng et al. used UiO-66 MOF loaded with Fe_3_O_4_ nanoparticles as the core and grew a triazine-based COF (TzDa-COF) on its surface through a one-pot process, producing a core–shell composite with a “Russian-doll” structure, Fe_3_O_4_@MOF-UiO-66@TzDa-COF [105]. This material exhibited ultrafast photocatalytic degradation performance toward the anionic dyes malachite green (MG) and Congo red (CR).

#### 2.2.2. In Situ Growth

The strategy of in situ growth facilitates the orientation of the nucleation and polymerization of COFs right on the surface of an already formed MOF core, allowing for the fabrication of core–shell MOF@COF composites that exhibit continuous structures and well-bonded interfaces. This method allows for meticulous management of the chemical and physical interactions that occur between the MOF core and COF shell. The interactions at these interfaces can be classified into two primary types: (1) Schiff base reactions, in which amine-aldehyde condensation forms robust C=N bonds that enhance interfacial stability [55,108]; (2) π–π stacking, which facilitates noncovalent interactions between aromatic groups, imparting flexibility and adaptability to the interface [109,110]. Together, these interactions overcome the limitations of single-component materials in terms of stability, tunable porosity, and functional diversity, which enhances the performance and expands the application possibilities in fields like gas adsorption, separation, and catalysis.

In a typical Schiff base reaction, the surface of the MOF is initially modified with amino groups. These -NH_2_ groups interact with aldehyde monomers, resulting in the formation of C=N imine linkages, which leads to the direct generation of COF nuclei on the MOF surface. Following this, the addition of more COF monomers facilitates the ongoing development of the COF layer atop the MOF, ultimately resulting in a distinct MOF@COF core–shell structure. In 2023, Li and colleagues reported the novel development of a tailored hydrophobic NTU-COF shell on MOF-5 by utilizing a “plug-socket anchoring” technique for the first time [106]. They applied a solvent-assisted ligand exchange method where H_2_BDC-NH_2_ substituted the external H_2_BDC linkers present on the surface of MOF-5. Following this, the aldehyde group from FPBA participated in a Schiff base reaction with the newly introduced -NH_2_ groups, leading to the polymerization of FPBA and TAPB on the MOF-5 surface, resulting in the formation of the NTU-COF shell (Figure 4d). The hydrophobic COF layer effectively protected MOF-5 from moisture and enabled excellent cycling performance for CO_2_/N_2_ separation under humid conditions. Zhang and colleagues created a core–shell Ti-MOF@TpTt structure through a sequential growth approach [107]. They chose MIL-125-NH_2_(Ti), recognized for its high thermal and chemical stability, as the MOF core and gradually incorporated 2,4,6-triformylphloroglucinol (Tp) alongside melamine (Tt) to form the TpTt-COF shell. The robust covalent bond connecting the Ti-MOF core with the TpTt COF shell, along with their advantageous bandgap and excellent light-harvesting ability, greatly improved the separation efficiency of photogenerated electron-hole pairs (Figure 4e). Following the modification with Pd, the Ti-MOF@TpTt demonstrated outstanding photocatalytic efficiency in the sequential reactions of hydrolysis of ammonia borane and hydrogenation of nitroarenes. In addition, Guo et al. utilized NH_2_-MIL-88B(Fe) as the core of the MOF and found that it was covalently bonded to the TpPa-1 COF via a Schiff base reaction, resulting in the creation of a core–shell NH_2_-MIL-88B(Fe)@COF composite [111]. When exposed to simulated sunlight, this material exhibited degradation efficiencies of 86% for tetracycline (TC) and complete degradation (100%) for rhodamine B (RhB). Peng et al. constructed a core–shell NH_2_-UiO-66@TpBD-COF composite by linking TpBD-COF onto the surface of NH_2_-UiO-66 via imine bonds [112]. This material achieved nearly quantitative conversion of benzylamine (94%) without the use of any sacrificial agent.

In constructing core–shell MOF@COF composites, in addition to Schiff base reactions between MOFs and COFs, the aromatic character of MOFs and the strong π-electron systems of COFs can drive ordered π–π stacking at the interface. This interaction promotes the spontaneous adsorption and alignment of COF monomers on the MOF surface, stabilizing the interface and facilitating the uniform growth of the COF shell. Xu et al. used Cu-MOF as the core and constructed a highly stable core-shell MOF@COF (Cu-MOF@CuPc-TA-COF) heterostructure through strong π–π stacking interactions [113]. Specifically, they used acetic acid as a catalyst and, under solvothermal conditions at 120 °C, introduced CuPc-TA and PDB, which were induced to grow on the surface of Cu-MOF, ultimately forming the core-shell Cu-MOF@CuPc-TA-COF. This core-shell structure not only retained the highly conjugated structure, abundant oxygen vacancies, mixed copper oxidation states (Cu^+^/Cu^0^), and large specific surface area of the original materials, but also exhibited enhanced photogenerated carrier separation. Wu and colleagues synthesized PCN-222-Cu@TpPa-1 by integrating a pre-synthesized COF into a solution containing a MOF precursor, resulting in a unique sunflower-like architecture stabilized through robust π–π interactions [114]. This novel material demonstrated exceptional catalytic performance for photocatalytic CO_2_ reduction without the need for sacrificial agents when exposed to simulated sunlight.

#### 2.2.3. Amorphous-to-Crystalline Transformation

During the synthesis of imine-based COFs, cross-linked amorphous polyimines typically form rapidly, resulting in low crystallinity or non-crystalline intermediates. Under specific thermodynamic conditions, these amorphous intermediates gradually transform into more ordered crystalline frameworks. The transition from a dynamic amorphous state to a crystalline one offers enhanced flexibility in synthesizing MOF@COF hybrid materials, allowing precise control over the process to produce composites with tailored structures and functionalities [115].

**Figure 5:**
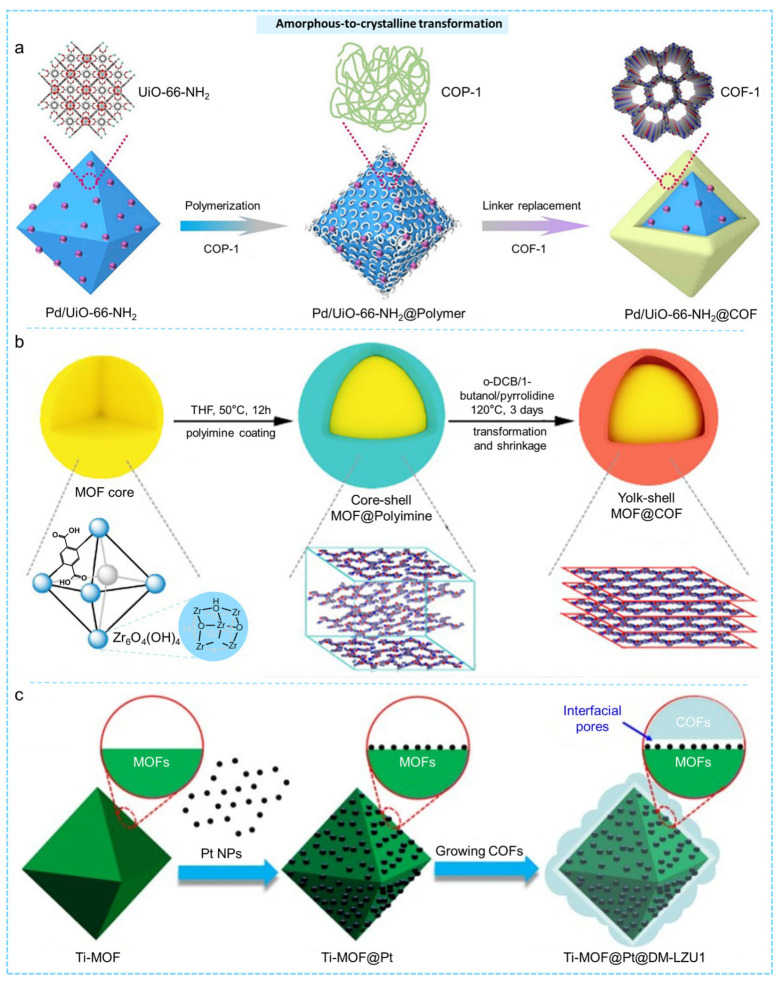
(**a**) Construction and preparation of sandwich-type Pd/UiO-66-NH_2_@COF composite structure [116]; (**b**) synthesis of yolk-structured MOF@COF nanocomposites and schematic diagram of in situ conversion of COF shell [117]; (**c**) preparation schematic diagram of Ti-MOF@Pt@DM-LZU1 composite material [118].

Zhou et al. utilized a strategy involving the transition from amorphous-to-crystalline states, with Pd/UiO-66-NH_2_ serving as the foundational core [116]. Initially, the Pd/UiO-66-NH_2_ was modified by introducing benzaldehyde, which was subsequently covalently bonded to 1,3,5-tris(4-amidophenyl)triazine, resulting in the formation of an amorphous covalent polymer (COP-1) on the surface of the MOF. They later substituted benzaldehyde with 2,5-dihydroxy-1,4-benzenedicarboxaldehyde to produce the core–shell composite Pd/UiO-66-NH_2_@COF-1 (Figure 5a). This material exhibited highly improved catalytic activity and substrate selectivity in olefin hydrogenation reactions. Similarly, Dang et al. used pre-synthesized UiO-66-(COOH)_2_ as the core and mixed it with the COF building blocks Tp and Pa to form a core–shell MOF@polymer composite with an amorphous polyimine shell (UiO-66-(COOH)_2_@Am-TpPa) (Figure 5b) [117]. The amorphous shell was transformed into a crystalline COF through a subsequent solvothermal treatment, resulting in the core–shell composite MOF@COF (UiO-66-(COOH)_2_@YS-TpPa), which demonstrated improved catalytic performance in a one-pot D-K tandem reaction due to synergistic effects. Moreover, this strategy was employed by Sun et al. to create core–shell Ti-MOFs@Pt@DM-LZU1, demonstrating outstanding photocatalytic performance when exposed to visible light irradiation (Figure 5c) [118]. Garzón-Tovar and colleagues successfully encapsulated MOF crystals within amorphous polymer microspheres made from imine-based TAPB-BTCA using a spray-drying technique [119]. Through dynamic covalent chemistry, the initially amorphous polymer underwent a transformation into crystalline COF-TAPB-BTCA, resulting in the creation of the core–shell structure UiO-66-NH_2_@COF-TAPB-BTCA. This composite demonstrated a level of porosity that significantly exceeded that of the separate MOF or COF components.

### 2.3. MOF@oxide

**Figure 6:**
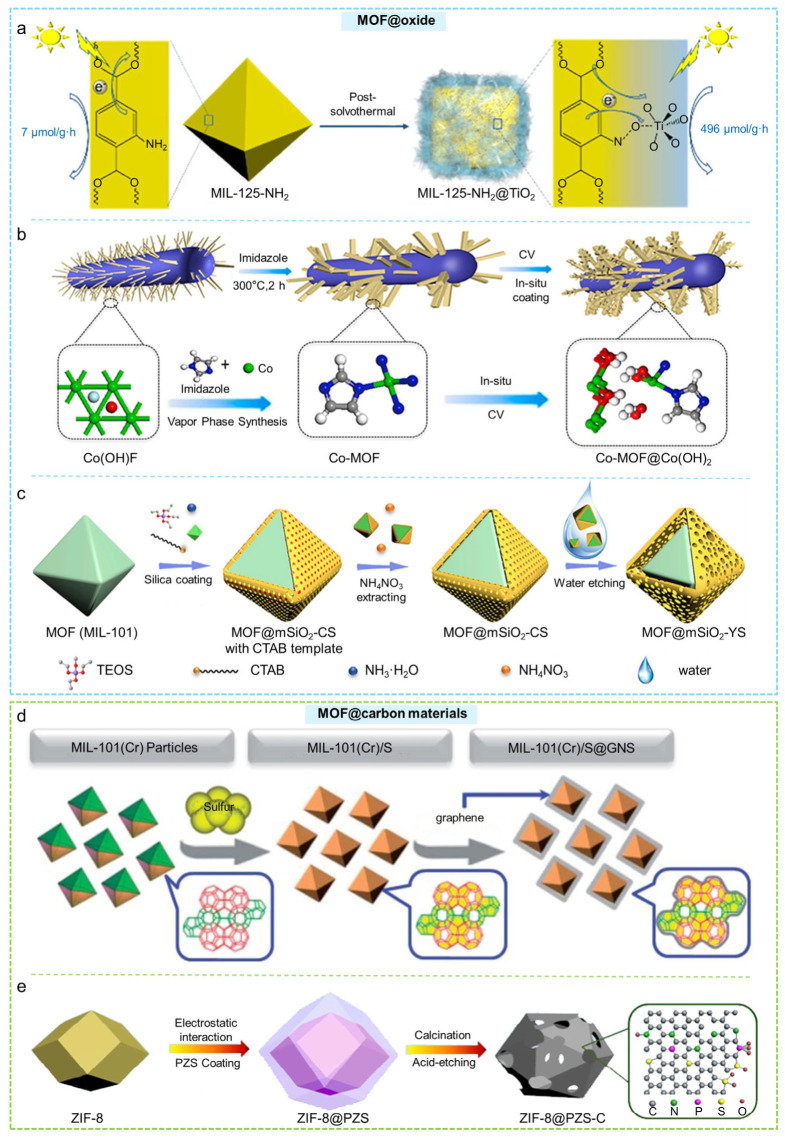
(**a**) Construction of MIL-125-NH_2_@TiO_2_ core–shell structure and its photocatalytic reaction diagram [121]. (**b**) Schematic illustration of the synthetic methods of Co-MOF@Co(OH)_2_ [57]. (**c**) Schematic representation of the preparation of yolk–shell MOF@mSiO_2_-YS nanoreactors [123]. (**d**) Preparation schematic diagram of MIL-101(Cr)/S@GNS composites [125]. (**e**) Schematic diagram of the preparation process of N, P, S co-doped hollow carbon polyhedron based on MOFs core–shell nanocomposites [126].

Core–shell MOF@oxide composites typically use a MOF as the inner core, while an oxide outer shell is constructed to achieve synergistic enhancements in structure and function. MOFs, with their highly ordered pore channels, tunable metal nodes, and organic linkers, serve as ideal structural templates and active site hosts during thermal treatment or post-synthetic modification [120]. In core–shell MOF@oxide composite materials, the oxide shell not only acts as a physical barrier that enhances the chemical stability of the MOF, but also optimizes electron transfer, surface properties, and mechanical strength through synergistic effects such as heterojunction formation, thereby improving their performance in applications including catalysis and separation. In 2018, Zhang and co-workers first introduced a post-solvothermal strategy to fabricate a core–shell MIL-125-NH_2_@TiO_2_ structure [121]. In this method, solvothermally synthesized MIL-125-NH_2_ powder was immersed in an ethanolic thioacetamide solution and subsequently heated at 200 °C for 2 h (Figure 6a). This treatment yielded MIL-125-NH_2_@TiO_2_ with a well-defined core–shell architecture. Owing to the abundant linker defects and oxygen vacancies generated during shell formation, the hydrogen evolution rate of MIL-125-NH_2_@TiO_2_ increased by approximately 70-fold compared with pristine MIL-125-NH_2_. Subsequently, the Li group employed a comparable post-solvothermal approach to create a core–shell composite, MIL-125@TiO_2_, resembling a marigold flower (MT-2) [122]. The resulting hierarchical design demonstrated outstanding photocatalytic efficiency in reducing Cr(VI) when exposed to radiation from a 300 W Xe UV lamp. Yao et al. employed an in situ cathodic electrodeposition approach to generate a Co(OH)_2_ coating on the surface of a Co-MOF, thereby constructing a core–shell Co-MOF@Co(OH)_2_ composite (Figure 6b) [57]. The resulting material exhibited remarkable oxygen evolution reaction (OER) activity along with excellent long-term operational stability.

In addition, Wang and co-workers used MIL-88B(Fe) as the core and adopted a Stöber method, employing CTAB as a structure-directing template to coat a SiO_2_ shell on the MOF surface, thereby forming the core–shell MIL-88B(Fe)@mSiO_2_ composite [58]. After subsequent calcination, the resulting yolk–shell nanoreactor (Fe/C@mSiO_2_) featured abundant iron active sites together with a protective mesoporous SiO_2_ shell, enabling the complete degradation of bisphenol A (BPA). Similarly, Bao et al. employed the Stöber method using MIL-101 as the core and CTAB as the mesostructural template to construct an mSiO_2_ shell on the MOF surface, yielding the core–shell MOF@mSiO_2_-CS material [123]. Subsequent selective water etching produced a yolk–shell nanoreactor (MOF@mSiO_2_-YS) (Figure 6c). The exposed active MOF core surface, the permeable mesoporous shell channels, and the mechanically protective rigid mSiO_2_ shell collectively endowed MOF@mSiO_2_-YS with enhanced stability in the CO_2_-epoxide (SO) cycloaddition reaction.

Wang and colleagues first proposed a solvent-dependent adsorption-driven mechanism [124]. In this strategy, ZIF-8 nanocrystals were pretreated by adsorbing CH_3_OH to obtain Pre-ZIF-8. Following this, in alkaline environments, the concurrent development of an mSiO_2_ shell along with the etching of the Pre-ZIF-8 core resulted in the creation of a core–shell ZIF-8@mSiO_2_ configuration. This material generated reactive oxygen species (ROS) under acoustic cavitation and exhibited outstanding sonodynamic performance.

### 2.4. MOF@carbon Materials

Core–shell MOF@carbon composites are composed of a MOF inner core and a carbon-based outer shell, which provides synergistic structural and functional advantages. Carbon shells can be generated via surface carbonization, polymer coating, or vapor deposition, significantly enhancing electrical conductivity, corrosion resistance, and overall structural robustness [127]. MOFs act as ideal templates, preserving their porous structure and active sites during the formation of the carbon shell, thus enabling high-performance catalytic and electrochemical applications. Therefore, constructing core–shell MOF@carbon composites with MOFs as the core and carbon materials as the shell can significantly enhance the overall structural robustness and environmental adaptability [128,129]. Zhao et al. first employed a melt-diffusion method to incorporate sulfur into MIL-101(Cr), yielding MIL-101(Cr)/S [125]. Graphene sheets (GNS) were then electrostatically coated onto the surface of MIL-101(Cr)/S, forming the core–shell MIL-101(Cr)/S@GNS composite (Figure 6d). As a cathode material, MIL-101(Cr)/S@GNS exhibited superior polysulfide-trapping capability and high electronic conductivity.

However, many porous MOFs are prone to hydrolysis and cannot be stored in air for extended periods due to their structural instability, as seen in materials such as HKUST-1 and MOF-5(IRMOF-1). Therefore, improving the moisture resistance of MOFs is crucial to promote their application under actual conditions. Yang and colleagues were the first to report that simple thermal treatment of IRMOF-1 at a specific temperature could coat the MOF with an amorphous carbon layer [130]. The resulting carbon-coated IRMOF-1 retained the original MOF’s morphology and pore structure while exhibiting significantly improved moisture stability.

Additionally, core–shell porous carbon materials derived from MOFs as templates or precursors exhibit excellent performance. Zhang et al. used ZIF-8 as a template and coated it with poly(cyclotriphosphazene-co-4,4′-sulfonyldiphenol) (PAD-C) to construct the core–shell ZIF-8@PAD-C [126]. Subsequent pyrolysis under an N_2_ atmosphere produced ZIF-8@Pd-C with a hierarchical hollow structure and robust electrical conductivity. In a NaCl solution with a concentration of 500 mg L^−1^, the ZIF-8@PZS-C electrode exhibited a considerable electrosorption capacity for salt, reaching 22.19 mg g^−1^ at a voltage of 1.2 V (Figure 6e). Subsequently, Yue and colleagues used Sn-MOF as a template and applied synchronously phosphor-sulfurizing resorcinol-formaldehyde coated Sn-MOF cubes, enabling the controlled synthesis of a unique porous core–shell SnPS_3_@C structure [131]. The resulting SnPS_3_@C featured a robust carbon protective layer, which enhanced the electrochemical activity and reduced polarization.

### 2.5. MOF@ionic Liquid/Polymer

Core–shell MOF@ionic liquid/polymer composites are constructed by coating the surface of MOF cores with a layer of ionic liquid or polymer, enabling a synergistic effect that combines structural protection with enhanced functional performance. Typical fabrication strategies include impregnation-crosslinking, surface polymerization, and vapor deposition, all of which enable the formation of a uniform and robust shell on the MOF surface. By combining the high surface area and adjustable porosity of MOFs with the functional characteristics of ILs or polymers, these core–shell structures notably enhance the structural stability and longevity of MOFs. Additionally, they provide advantageous attributes, including improved gas adsorption selectivity, heightened catalytic effectiveness, and improved biocompatibility. ILs are salts that remain liquid at or near room temperature. They have tunable structures, task-specific functions, and strong affinity for many molecules [132,133]. When ionic liquids are introduced into core–shell MOF structures, the porous framework of MOFs is combined with the adjustable physicochemical properties of ionic liquids. This combination improves selective transport as well as adsorption and catalytic performance. Polymers show good flexibility, thermal stability, and chemical resistance. Functional polymer shells can be designed around high-porosity MOF cores [134]. These composites remain stable under moisture, acidic or basic conditions, and thermal stress. The polymer shell also provides selective molecular sieving and controlled mass transport [135,136]. Functional groups in the shell can adjust hydrophilicity, charge distribution, and the local reaction environment, which improves adsorption, catalysis, separation, and sensing behavior.

#### 2.5.1. Impregnation-Crosslinking Method

**Figure 7:**
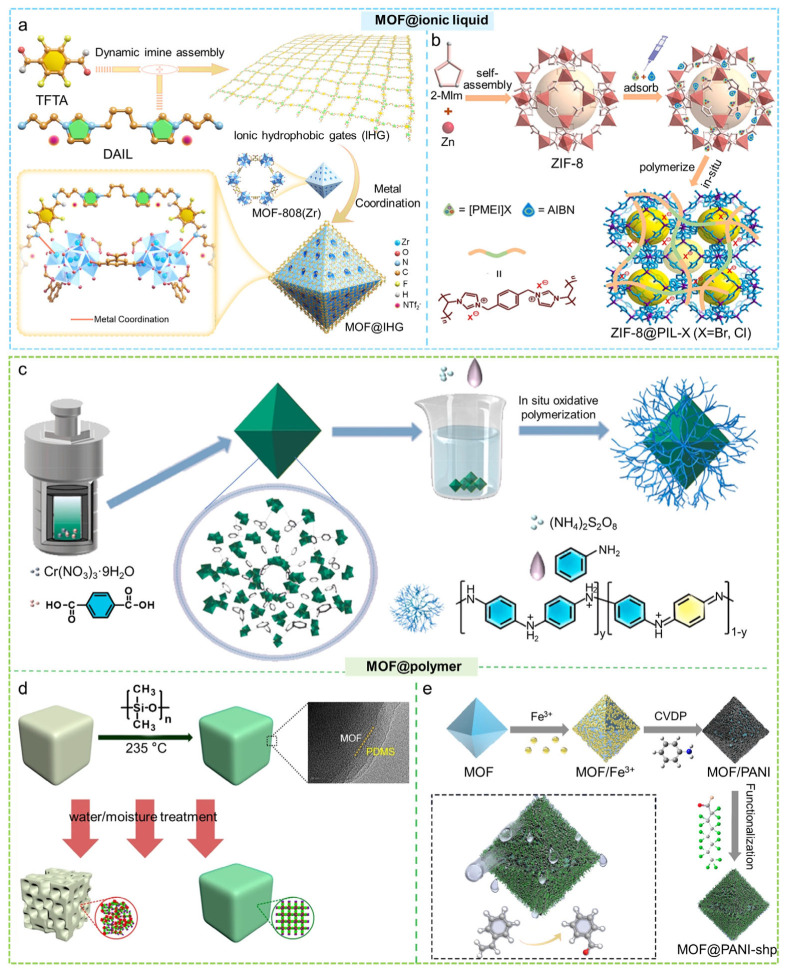
(**a**) Schematic illustration of the supramolecular construction of the MOF@IHG composite [137]; (**b**) construction of ZIF-8@PIL-X (X = Br, Cl) composites [141]; (**c**) preparation flow chart of M@PANI-X [142]; (**d**) synthesis process of PDMS coating on the surface of MOFs and the improvement of moisture/water resistance of MOFs [143]; (**e**) Synthesis diagram of MOF@PANI-shp prepared by post-synthesis polymerization modification method [144].

The impregnation-crosslinking method has become one of the commonly used and efficient strategies for preparing core–shell MOF@ionic liquid/polymer composites, due to its simple operation, mild reaction conditions, and good universality. Our research group developed an innovative hydrophobic ionic liquid gating strategy on MOF cores [137]. First, a Schiff base reaction was used to covalently assemble a hydrophobic ionic liquid with tetrafluoroterephthalaldehyde (TFTA), forming a hydrophobic ionic liquid polymer (IHG). The IHG was then assembled onto the surface of MOFs via metal coordination, producing a core–shell MOF@IHG composite that simultaneously provides a water penetration barrier and CO_2_-selective transport channels, enabling the direct capture of high-purity CO_2_ from humid flue gas in a single step (Figure 7a). Furthermore, Liu and colleagues prepared a core–shell [TETA]L@ZIF-8@[TETA]L structure via impregnation [138]. This configuration features an outer IL shell, a central ZIF-8 layer, and an inner IL core. Such a design provided the material with a significant capacity for CO_2_ adsorption and outstanding separation capabilities, resulting in a CO_2_/CH_4_ selectivity ranging between 260 and 1990, and CO_2_/N_2_ selectivity of 1688–5572. Zhang et al. used a simple impregnation approach to synthesize a bifunctional core–shell MOF@POP (MIL-101(Cr)@PMF) composite material, with MIL-101(Cr) as the core and polymelamine formaldehyde (PMF) as the shell [139]. The material produced exhibits both Brønsted base sites and Lewis acid sites, demonstrating outstanding catalytic performance in the deacetalization–Knoevenagel tandem reaction. In order to shield MOFs from water molecule intrusion and improve their stability in aqueous environments, Qian and colleagues utilized a solution impregnation technique to apply DC 1-2577 (organosilicone) onto the surfaces of the MOFs [140]. This approach led to the development of core–shell SH MOFs, incorporating NH_2_-MIL-125(Ti), ZIF-67, and HKUST-1. The hydrophobic layer that developed on the surface of the MOF not only greatly enhanced their stability in water but also offered enduring protection against degradation caused by water.

#### 2.5.2. Surface Polymerization

Surface polymerization is one of the commonly used methods for constructing core–shell MOF@ionic liquid/polymer composites. In this approach, functional monomers are first polymerized with a crosslinker in solution to form a polymer precursor. The polymer is then assembled onto the MOF surface through metal coordination or surface chemical interactions, forming a core–shell structure. This method preserves the MOF’s open channels while providing features such as a water permeation barrier or selective gas transport pathways, enhancing the composite’s performance in gas separation, catalysis, and mass transfer applications. Dong et al. for the first time employed an in situ polymerization strategy to coat 1,1′-[1,4-phenylenebis(methylene)]bis[3-ethenylimidazolium] ([PMEI]Br) onto the ZIF-8 surface, constructing a core–shell ZIF-8@poly(ionic liquids) composite (Figure 7b) [141]. Through meticulous regulation of the polymerization monomer composition, the resulting poly(ionic liquids) created a strong polymer network characterized by a high concentration of Br^−^ sites on the surface of ZIF-8, thereby significantly improving the structural stability of ZIF-8. The core–shell structure of ZIF-8@poly(ionic liquids) demonstrated remarkable catalytic effectiveness, recyclability, and an extensive range of substrates for the production of cyclic carbonates without additives under mild conditions. Ci et al. utilized the microporous characteristics of MIL-101(Cr) to adsorb aniline, which was subsequently followed by oxidative polymerization occurring on the surface of the core [142]. The product of this process was a core–shell MOF@polyaniline composite, which showed enhanced Cr(IV) adsorption kinetic and higher sorption capacity (Figure 7c). In similar work, Huang et al. developed a nonsolvent-induced surface-directed polymerization strategy to fabricate core–shell MOF@polymer materials having MOF-801 as the core and a polyamide, polyurea, or polythiourea as the shell [145]. Observation from the TEM showed that the formed polymer shells were between 2 and 10 nm in thickness. The MOF-801 coating’s polymers improved the mechanical strength under harsh conditions. The MOF@HDA-PDTC exhibited a significantly higher Hg(II) removal efficiency than the corresponding pure polymer. A rapid polymerization method was developed by Wang and co-workers for encapsulating the homogeneous catalyst phosphotungstic acid (PTA) within MIL-101(Cr) [146]. Through a nonsolvent-induced surface-directed polymerization strategy, the researchers added a nonsolvent into the reaction mixture to induce phase separation, causing the monomers to preferentially concentrate on the MOF surface. This locally enriched monomer environment greatly accelerated the polycondensation between dianhydride and polyamine monomers, enabling the formation of a dense, nonporous polymer coating on the MOF exterior within a short time. The core–shell MOF@polymer material demonstrated impressive catalytic effectiveness in the oxidative breakdown of phenol.

#### 2.5.3. Vapor Deposition

Vapor deposition is a common and effective method to construct hydrophobic polymer coatings on the surface of MOFs. Zhang’s group modified MOFs with hydrophobic polydimethylsiloxane (PDMS) using a simple vapor deposition technique, which markedly enhanced the moisture resistance and water stability of the MOF materials (Figure 7d) [143]. Zhang and colleagues synthesized a composite of polyaniline (PANI) and MOFs using vapor-phase polymerization, followed by the covalent attachment of hydrophobic molecules to PANI [144]. This process produced a superhydrophobic core–shell composite of MOF@PANI. The available active sites and the synergistic properties of this material provide it with a unique catalytic selectivity for the oxidation of styrene (Figure 7e).

### 2.6. A@MOF

By introducing carbon materials, oxides, nanoparticles, and other components into MOFs, the resulting core–shell A@MOF structures can achieve a more effective synergistic integration of multiple material properties compared with analogous MOF@MOF, MOF@COF, or MOF@polymer architectures [147]. As the primary framework, the MOF not only provides a high specific surface area and a uniform, tunable pore network but also offers a naturally compatible spatial environment for anchoring and distributing guest components. Benefiting from the confinement effect of the MOF pore size and its unique coordination microenvironment, the incorporated guest materials are less prone to migration, aggregation, or deactivation within the pores, which significantly enhances the structural and cycling stability of the entire system. In terms of synthesis strategies, the construction pathways for A@MOF composites are diverse, including encapsulating pre-synthesized structures within the MOF, in situ synthesis, and impregnating functional groups into the MOF. These approaches provide abundant possibilities for the efficient fabrication and structural design of these composite materials.

**Figure 8:**
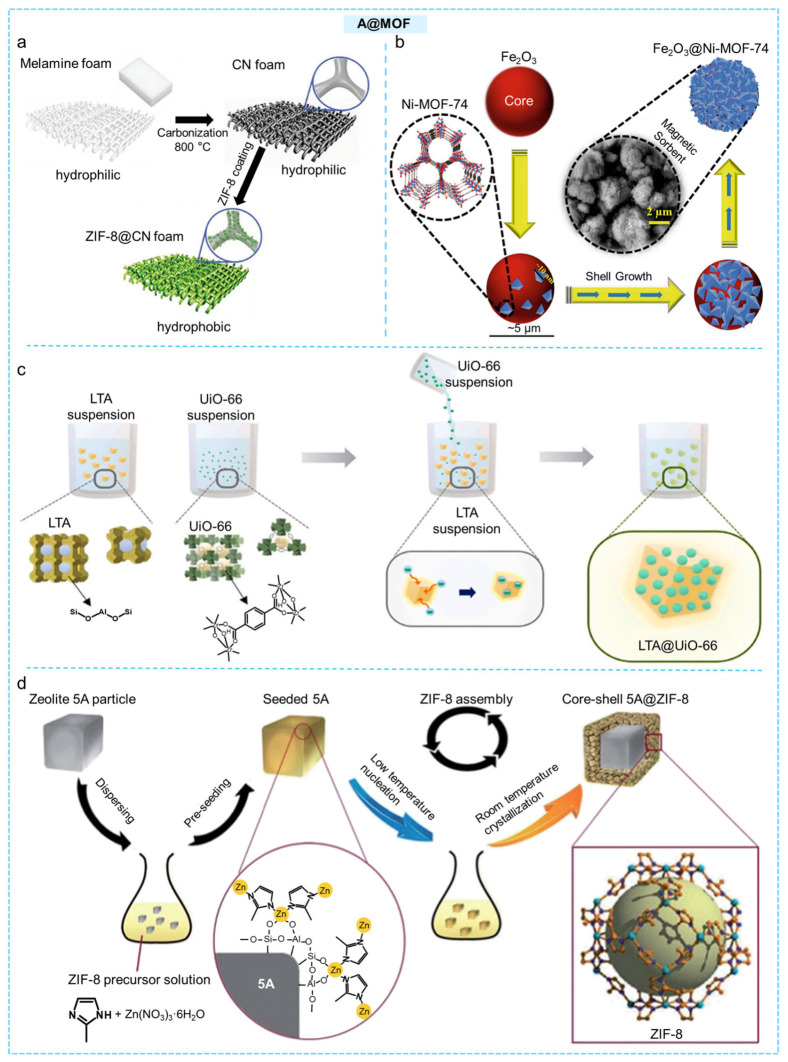
(**a**) Synthesis schematic of hierarchical porous ZIF-8@CN foams [149]; (**b**) schematic demonstration of Fex@MOF composites synthesis [150]; (**c**) schematic diagram of the synthesis of LTA@UiO-66 particles [151]; (**d**) synthesis diagram of 5A@ZIF-8 [152].

Lei and co-workers employed an in situ coating strategy, introducing 5,10,15,20-tetrakis(4-sulfonatophenyl) porphyrinato iron (III) chloride (FeTPPs) into the cages of Cu-BTC through a one-pot method to successfully prepare an adsorbent (FeTPPs@Cu-BTC) for CO_2_ separation [148]. The sulfonate groups provided additional CO_2_ adsorption sites, and due to their weak coordination ability, they effectively prevented FeTPPs from coordinating with Cu^2+^. The resulting FeTPPs@Cu-BTC exhibited outstanding separation performance, with CO_2_/N_2_ and CO_2_/CH_4_ selectivities of 57.7 and 14, respectively. Kim et al. rapidly carbonized melamine foam to obtain a graphitic carbon nitride (CN) foam template, which was then immersed in a solution containing ZIF-8 precursors to allow in situ growth and form a hierarchical core–shell CN@ZIF-8 structure (Figure 8a) [149]. The growth of ZIF-8 altered the initially hydrophilic surface of the CN foam to a highly hydrophobic state, achieving a water contact angle of 135°. This modified material was able to selectively absorb as much as 58 wt% of oil from water–oil mixtures and effectively facilitate the transformation of CO_2_ into chloropropylene carbonate. Baamran and co-workers used Fe_2_O_3_ as the core and grew Ni-MOF-74 in situ on its surface via a hydrothermal method to prepare the core–shell magnetic adsorbent Fe_2_O_3_@Ni-MOF-74 (Figure 8b). They further evaluated its separation performance for C_2_H_4_/C_2_H_6_ [150]. Tikue’s team utilized the difference in surface zeta potential to promote the formation of MOFs on hydrophilic LTA zeolite, employing LTA as the central component to create a sturdy core–shell LTA@UiO-66 structure (Figure 8c) [151]. When incorporated into polyimide to fabricate a mixed matrix membrane (LTA@UiO-66/PI), the resulting membrane exhibited exceptional mechanical integrity and outstanding gas separation performance, with O_2_/N_2_ selectivity of 10.7 and CO_2_/CH_4_ selectivity of 47. Similarly, Gao and co-workers combined a prenucleation step with a two-stage temperature-controlled crystallization process to synthesize the core–shell 5A@ZIF-8 composite material using 5A zeolite as the core (Figure 8d) [152]. During the separation of simulated wet flue gas (15% CO_2_, 90% humidity, 298 K), the composite exhibited a dynamic hydrophobic hindrance effect, achieving a CO_2_ adsorption capacity as high as 2.67 mmol g^−1^ and a CO_2_/H_2_O selectivity of 6.61. Cho et al. used Cu-BTC as a sacrificial template and introduced thioacetamide into the MOF via a simple solution infiltration method to prepare the nano-CuS@Cu-BTC composite [153]. By increasing the CuS content in the material, the electrical conductivity was enhanced by 10^9^ times compared with that of Cu-BTC. Liu et al. encapsulated C_60_ into NU-901 via an adsorption strategy, successfully constructing a novel core–shell MOF-based photocatalyst, C_60_@NU-901. Owing to the host–guest interactions and the inhomogeneous charge distribution, a substantial electrostatic potential difference was generated within C_60_@NU-901, leading to a strong built-in electric field. As a result, the photocatalytic activity of C_60_@NU-901 is 10.7 times higher than that of NU-901 [154]. Similarly, Niu et al. encapsulated C_60_ into the cavities of [La(BTB)]_n_ through π–π stacking constructing a chiral core–shell composite, C_60_@[La(BTB)]_n_. The host–guest interactions and the inhomogeneous charge distribution generate a strong built-in electric field, thereby effectively enhancing the electrical conductivity of the chiral material [155].

To facilitate a clearer comparison of the structural features and functional advantages of the different types of core–shell MOF-based composites discussed above, Table 1 summarizes representative examples of MOF@MOF, MOF@COF, MOF@oxide, MOF@carbon, MOF@ionic liquid/polymer, and A@MOF systems. The table focuses on changes in the surface area and pore size before and after core–shell construction and, where available, provides approximate values for core dimensions and shell thicknesses. It can be seen that the introduction of a shell component can substantially modify the pore structure and surface area of the parent MOF, while the sizes of the core and shell vary widely depending on the composite type and synthesis approach. These structural variations are closely associated with the performance of the materials in applications such as gas adsorption and separation, catalysis, energy storage, and sensing.

## 3. Applications of Core–Shell MOF Composites

Notably, core–shell MOFs and their derived materials exhibit unique structural advantages, such as controllable core–shell interfaces, high specific surface areas, abundant metal sites, and tunable pore architectures, which confer broad applicability in catalysis, adsorption and separation. This part of the paper highlights the application of core–shell MOF composites in different fields, and their role in addressing futuristic scientific and technological tasks. The next subsections will discuss the applications in catalysis, adsorption and separation, demonstrating their usability and versatility.

### 3.1. Catalytic Reaction

#### 3.1.1. Thermocatalytic Reaction

Core–shell MOFs have controllable core–shell structures and distinctive interface designs which can produce novel properties in thermal catalysis. The MOF core possesses a substrate molecule enrichment and selective adsorption capability, whereas the shell distinctly alters the diffusion rate of reactant molecules via pore size modulation and chemical functionalization, thereby improving the selectivity and enhancing the conversion efficiency. Moreover, electronic or spatial effects at the core shell interface, such as the synergistic interactions between metal ions or the modulation of the core electronic environment by the shell, can accelerate key reaction steps or alter reaction pathways. As a result, core–shell MOFs demonstrate higher catalytic activity than single-component MOFs and exhibit outstanding performance, selectivity, and stability in thermal catalytic reactions including oxidation, hydrogenation, cycloaddition, and condensation.

**Figure 9:**
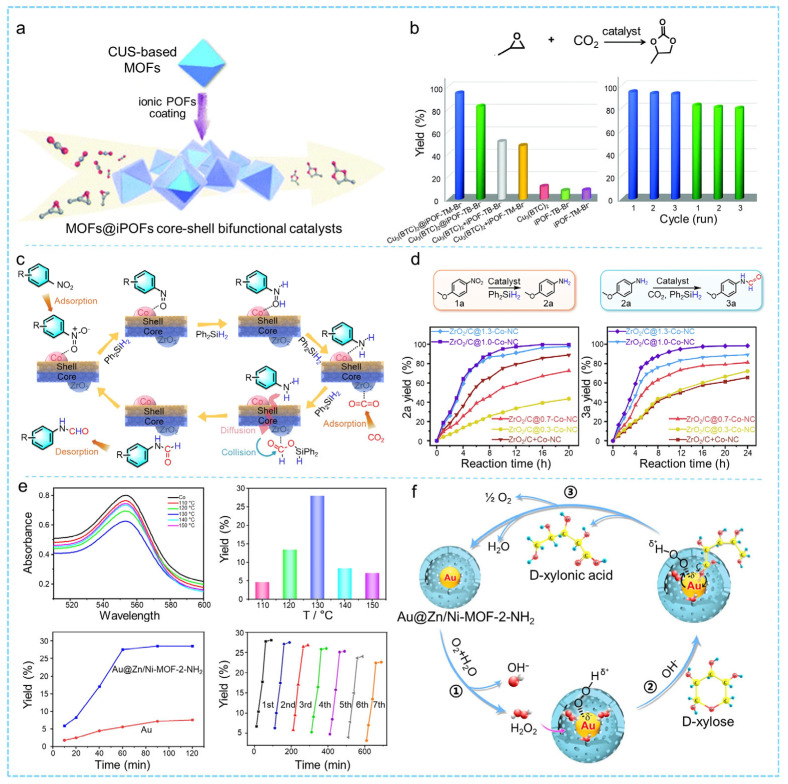
(**a**) Illustration of the catalytic concept of core–shell MOF@iPOF [161]; (**b**) catalytic conversion of CO_2_ cycloaddition with PO over different catalysts; recycling performance of Cu_3_(BTC)_2_@iPOF-TB-Br^−^ (green) and Cu_3_(BTC)_2_@iPOF-TM-Br^−^ (blue) in three consecutive runs of CO_2_ cycloaddition [161]; (**c**) catalytic process of ZrO_2_/C@x-Co-NC for one-pot tandem conversion of CO_2_ and nitroaromatics; (**d**) together with the time-dependent product yields in the above reactions [89]; (**e**) catalytic performance and (**f**) reaction mechanism of D-xylose oxidation over hollow Au@Zn/Ni-MOF-2-NH_2_ (① Adsorption and activation; ② Ring-opening and alkoxide formation; ③ Dehydrogenation and oxidation) [164].

CO_2_ serves as a plentiful, cost-effective, and non-toxic feedstock for C1 that can be transformed into cyclic carbonates via its reaction with epoxides in a cycloaddition process [156,157]. Given the significant thermodynamic and kinetic stability of CO_2_, efficient and recyclable heterogeneous catalysts are required to enable this transformation under mild conditions [158,159]. Certain MOFs contain numerous unsaturated metal sites and can function as Lewis acid catalysts to promote the cycloaddition of CO_2_ and epoxides, but they typically require additional co-catalysts [160]. By using a MOF as the core to construct bifunctional core–shell MOF catalysts, efficient CO_2_ cycloaddition can be achieved without any co-catalyst. This design also preserves open channels and enhances mass transfer and catalytic efficiency. Liu and co-workers used an ionic porous organic framework (POF) as the shell material to construct two core–shell catalysts, Cu_3_(BTC)_2_@iPOF-TB-Br^−^ and Cu_3_(BTC)_2_@iPOF-TM-Br^−^ [161]. Owing to the synergistic cooperation between the dual active sites, these catalysts exhibited outstanding performance in the CO_2_ cycloaddition reaction under mild, co-catalyst-free conditions (60 °C, 0.5 MPa CO_2_, 24 h) (Figure 9a,b). Liu and co-workers embedded NH_2_-UiO-66 as an “adsorption” engine within an ionic organic polymer (iCOP) shell to construct the core–shell catalyst NH_2_-UiO-66@iCOP [162]. The ionic active centers on the iCOP shell, the Lewis acidic Zr sites of the MOF core, and the synergistic effects at the core–shell interface collectively enabled the efficient conversion of CO_2_ into cyclic carbonates under mild, co-catalyst-free conditions, achieving product yields of 90–99% for various cyclic carbonates, among which the yield of chloropropene carbonate with NH_2_-UiO-66@iCOP is approximately 7.5 times higher than that of the NH_2_-UiO-66 core alone. In addition, Zhao and co-workers synthesized a core–shell NH_2_-UiO-66(Zr)@COF featuring abundant Lewis acid sites and pronounced π–π stacking interactions, which endowed the material with excellent CO_2_ adsorption capacity and selectivity [163]. Through simple post-synthetic modification, an ion-functionalized derivative with active side chains, NH_2_-UiO-66(Zr)@COF-0.15-Br, was prepared. The introduced Br^−^ species effectively promoted reactant activation and lowered the reaction energy barrier, thereby facilitating the efficient conversion of CO_2_ into both monocyclic and polycyclic carbonates.

The hydrogenation reaction refers to the catalytic conversion of unsaturated bonds (such as C=N, C=O, or C≡C) into their corresponding saturated products by reacting with hydrogen gas [165,166]. The reaction generally proceeds through three key steps: dissociation of H_2_ on the catalyst surface to form active hydrogen species, adsorption of the substrate molecule onto the catalyst, and subsequent migration of active hydrogen atoms to the substrate to complete the hydrogenation process. The reaction rate and selectivity are largely governed by the nature of the catalyst, the structure of the substrate, and the specific reaction conditions. A bifunctional core–shell catalyst, designated as ZrO_2_/C@x-Co-NC, was developed by Qin’s team through the pyrolysis of a ZIF-67@UiO-66 heterostructure [89]. In this system, Co nanoparticles and ZrO_2_ act as highly efficient sites for the hydrogenation process of nitroarenes and the following N-formylation of the produced aromatic amines using CO_2_, respectively (Figure 9c). Notably, the optimized core–shell catalyst achieved an unparalleled yield of 99.5% for *N*-(4-methoxyphenyl)formamide in the one-pot cascade hydrogenation–formylation reaction of 4-nitroanisole with CO_2_, which is significantly higher than those obtained with ZrO_2_/C (0%), Co-NC (14.1%), and the physically mixed catalyst (52.3%) (Figure 9d). Li et al. prepared a core–shell Ni_x_Fe@C catalyst by pyrolyzing Fe/Ni-MOF-74 for the hydrogenation of CO_2_ to ethylene [167]. Their study revealed that the incorporation of an appropriate amount of Fe enhances metal dispersion and improves the CO_2_ adsorption capacity. Among them, the Ni_7_Fe@C catalyst attained a CO_2_ conversion rate of 53.3% at 300 °C, double that of the Ni@C catalyst alone. Moreover, Chen and colleagues utilized activated hydrogen atoms as reducing agents to facilitate the targeted deposition of Ag onto Pd, thus producing ultrafine Pd@Ag core–shell nanoparticles within the pores of the MOF [168]. Owing to the surface electronic modulation of Pd active sites by Ag, the resulting Pd@Ag core–shell nanoparticles exhibited outstanding selectivity in the hydrogenation of phenylacetylene. Furthermore, the Co/MnO_x_@quasi-MOF-74 catalyst exhibits three synergistic active sites (Co^0^, Co^2+^, and Co_2_C) that facilitate the conversion of syngas (CO/H_2_) into higher alcohols (C_2_+OH) by effectively promoting CO dissociation, coupling of CH_x_-CH_y_, and CO insertion, thereby leading to a substantial increase in alcohol yields [169]. Under conditions of 200 °C and 3.0 MPa, the catalyst achieved a total alcohol selectivity of 48.7 wt%, with C_2_+OH accounting for 93.2 wt%, minimal CH_4_ byproduct, and undetectable CO_2_. Its catalytic performance is comparable to that of multifunctional catalytic systems operating under high-pressure conditions.

Oxidation reactions, as a major category in thermal catalysis, play a pivotal role in energy conversion, pollution control, and the synthesis of high-value chemicals. These reactions generally involve the transfer of oxygen atoms from an oxidant (such as O_2_, H_2_O_2_, or metal oxides) to a substrate, resulting in electron loss and the structural transformation of the substrate. Representative thermal catalytic oxidation processes include the selective oxidation of hydrocarbons, CO oxidation, degradation of volatile organic compounds (VOCs), and oxidative upgrading of alcohols and aldehydes. Ying et al. coated MIL-101(Cr) with a SiO_2_ shell to obtain MIL-101(Cr)@mSiO_2_, which significantly improved both the catalytic stability and catalytic activity of the material in the oxidation of indane, retaining 82% of the initial conversion, which is markedly higher than that of MIL-101Cr (46%) [170]. Qin et al. prepared a core–shell Au@Zn/NiMOF-2-NH_2_ catalyst, in which ultrafine Au nanoparticles were well dispersed within the hollow, hierarchically structured, amino-functionalized bimetallic MOF shell [164]. Due to the collaborative effect of the Au nanoparticles and the hollow shell of the MOF, the obtained nanocatalyst demonstrated outstanding catalytic performance for the oxidation of D-xylose in the absence of a base, reaching a turnover frequency (TOF) of up to 76.53 h^−1^, which is 306 times greater than that of the unmodified Au nanoparticle catalyst (Figure 9e,f).

Condensation reactions are typically driven by thermal energy, in which catalysts facilitate intermolecular or intramolecular bond formation and the elimination of small molecules through the synergistic action of acidic and basic active sites, thereby efficiently constructing more complex organic structures. Gao et al. developed a bifunctional catalyst, MOF@COF (PCN-222-Co@TpPa-1), by utilizing strong π–π stacking interactions [171]. The catalyst features Lewis acidic sites (Co(II) centers and Zr(IV) clusters) and Brønsted basic sites (imine groups) within the TpPa-1 shell. It demonstrated impressive catalytic activity, achieving a yield of 99.3%, and showed robust recyclability during the one-pot deacetalization–Knoevenagel condensation cascade reaction.

Overall, different types of core–shell MOF composites exhibit distinct suitability in thermocatalytic reactions. MOF@MOF systems, benefiting from good lattice matching and continuous pore connectivity between the core and shell, are generally more suitable for reactions that require efficient mass transport and high structural stability, such as CO_2_ cycloaddition and selective oxidation. MOF@COF architectures, enabled by the excellent chemical tunability of COF shells, strong π–π interactions, and the integration of multiple functional sites, show particular advantages in thermocatalytic processes involving acid–base cooperation or multistep reaction pathways. In contrast, MOF@ionic liquid/polymer systems rely on flexible chains and ionic or polar functional groups to regulate the local reaction microenvironment and enhance substrate enrichment, leading to outstanding activity and selectivity in CO_2_ conversion reactions. Meanwhile, MOF-derived carbon-based core–shell composites offer high thermal stability and efficient electron transport, and the encapsulated or supported metal/metal oxide active sites are especially favorable for hydrogenation reactions, where interfacial electronic effects and metal synergism can significantly enhance catalytic activity and selectivity. Accordingly, the rational selection of MOF cores and shell materials tailored to specific thermocatalytic reactions is a key design principle for achieving high catalytic performance.

#### 3.1.2. Electrocatalytic Reaction

In recent years, core–shell MOF composites have been successfully applied in key electrocatalytic processes such as the oxygen reduction reaction (ORR), hydrogen evolution reaction (HER), OER, and electrochemical CO_2_ reduction reaction (ECO_2_RR) [172]. Core–shell MOF catalysts demonstrate promising catalytic performance and durability in metal–air batteries and fuel cells. Moreover, they display significant selectivity and effectiveness in the conversion of CO_2_ and in reactions involving the oxidation of small molecules. Additionally, optimizing the types of metal centers, modifying organic linkers, and adjusting the material structure can enhance the catalytic performance, establishing core–shell MOFs as a crucial foundation for developing high-performance catalysts in electrocatalysis.

The OER and ORR are two closely related core processes in electrocatalysis. These processes generally take place at the anode and cathode, respectively, within systems such as water electrolysis, fuel cells, and metal–air batteries [173]. OER is the oxidation of water or hydroxide, involving multi-electron transfer to generate O_2_. Its sluggish kinetics and high overpotential place stringent demands on the oxidative stability and activity of the catalyst [174]. ORR, in contrast, is the reduction in O_2_, proceeding through either a 2e^−^ or 4e^−^ pathway to form H_2_O or OH^−^. Effective ORR catalysts require high activity, high selectivity, and excellent electron and proton transport properties [175]. In recent years, core–shell MOFs, with their tunable structures and rich interfacial characteristics, have been widely employed in developing highly efficient electrocatalysts for both OER and ORR.

**Figure 10:**
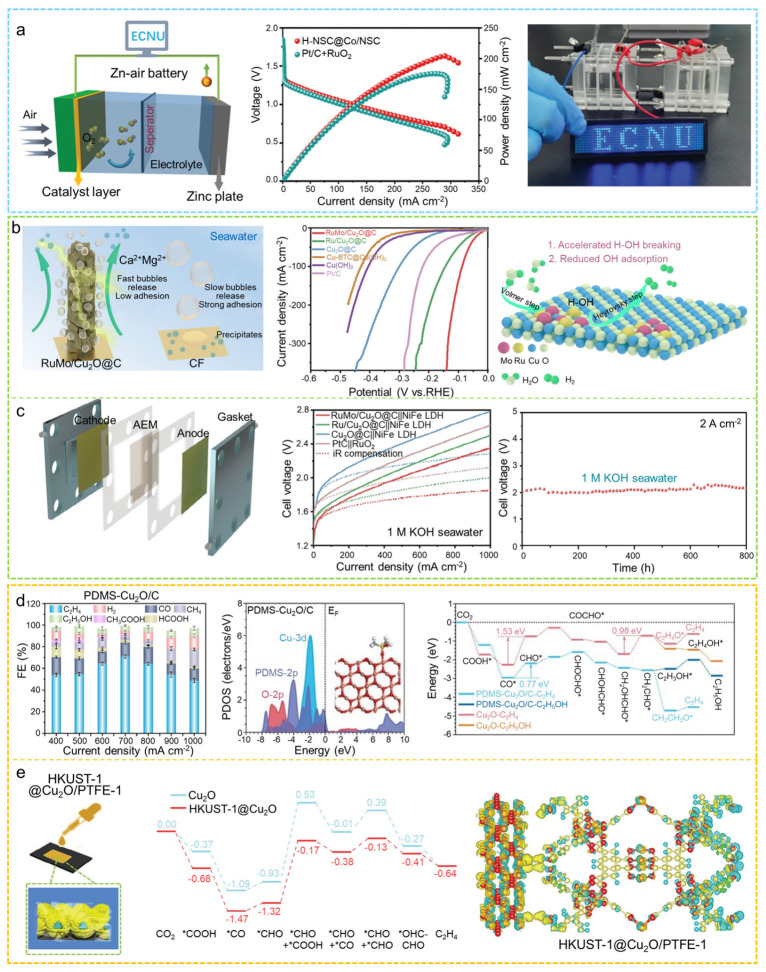
(**a**) Configuration diagram of rechargeable zinc–air battery, and discharge polarization curves and power density graphs for H-NSC@Co/NSC and Pt/C+RuO_2_-based zinc–air batteries, and a blue LED illuminated by two ZRBs connected in series [177]; (**b**) schematic representation of the behavior of underwater bubbles on the surfaces of RuMo/Cu_2_O@C and CF, including HER polarization curves for various catalysts in a 1 M KOH electrolyte (accounting for an IR compensation of 1.42 ± 0.11 Ω), as well as the suggested mechanism for the improved HER performance of RuMo/Cu_2_O@C (The dashed lines represent the LSV polarization curves with IR compensation, where red corresponds to condition RuMo/Cu_2_O@C||NiFe LDH, green to condition Ru/Cu_2_O@C||NiFe LDH, blue to condition Cu_2_O@C||NiFe LDH, and pink to condition PtC||RuO_2_) [183]; (**c**) schematic illustration of the AEM device, along with the polarization curves for various materials in 1.0 M KOH seawater, and the stability curve of RuMo/Cu_2_O@C||NiFe LDH in an alkaline electrolyzer operating at a current density of 2 A cm^−2^ in 1.0 M KOH seawater [183]; (**d**) the FEs and PDOS of PDMS-Cu_2_O/C, and the CO_2_RR reaction trends of Cu_2_O and PDMS-Cu_2_O/C [185]; (**e**) preparation of HKUST-1@Cu_2_O/PTFE-1 gas diffusion layer, DFT-calculated Gibbs free energy of the Cu_2_O and HKUST-1@Cu_2_O catalysts, and charge density distribution of HKUST-1@Cu_2_O [186].

Zhou et al. used a core–shell Zn-MOF@Co-MOF as a self-templating agent to prepare a bifunctional Co/CoO@NSC catalyst, which exhibited greater electrocatalytic activity for both OER and ORR comparable to that of Pt/C+RuO_2_ [176]. The Co/CoO@NSC-based aqueous Zn–air battery delivered a high specific capacity (759.7 mAh g^−1^), high energy density (990.5 Wh kg^−1^), and exceptionally long cycling stability (over 400 h/1200 cycles). Li and co-workers successfully prepared a core–shell electrocatalyst, H-NSC@Co/NSC, by pyrolyzing COF@ZIF-67 [177]. The resulting catalyst possesses abundant active sites and exhibits superior OER and ORR performance, along with a small potential gap (ΔE = 0.75 V). The Zn–air battery assembled with H-NSC@Co/NSC delivers a high power density of 204.3 mW cm^−2^ and demonstrates stable rechargeability (Figure 10a). Zhang and colleagues fabricated yolk–shell hollow polyhedra (YHPs) containing Ni-Co-Fe ternary alloys and metal oxides by controllably etching multilayer ZIF structures followed by pyrolysis [178]. This architecture provides efficient mass transfer channels, good electrical conductivity, and full exposure of internal active centers. Leveraging the combined advantages of the alloy, oxide species, and the hollow yolk–shell architecture, YHP-1 displays remarkable performance in the ORR, achieving a half-wave potential of 0.79 V. In contrast, YHP-2 shows impressive activity for the ORR, necessitating an overpotential of merely 257 mV at a current density of 10 mA cm^−2^. Additionally, Xu et al. constructed a two-dimensional MOF with a multilayer shell structure and obtained a Co/Ni-embedded N-doped porous carbon material (Ni/Co-NC) after high-temperature pyrolysis [179]. The synergistic effect between Ni and Co, the interfacial characteristics of the core–shell structure, the 2D morphology, and the exposed active sites collectively endow the material with ORR performance comparable to Pt/C. In addition, it demonstrated remarkable electrochemical capabilities in zinc–air batteries, achieving a peak power density of up to 243.4 mW cm^−2^, more than double that of the traditional Pt/C+RuO_2_ catalyst system (106.9 mW cm^−2^).

Considered one of the most promising alternative energy sources, hydrogen energy is valued for its clean and renewable characteristics. Water electrolysis has emerged as a key method for production, thanks to its sustainability and the high purity of the hydrogen generated [180,181]. However, the intrinsically slow kinetics of the HER require highly efficient and stable electrocatalysts. Although Pt exhibits the best HER activity, its high cost and limited reserves have driven researchers to explore low-cost alternatives [182]. In recent years, core–shell MOFs and their derived composites have gained significant attention, as they allow the precise tuning of active metal center distribution, optimization of pore structures, and construction of multilevel and multiphase heterointerfaces [183,184]. After pyrolysis, these materials can provide high surface areas, porous frameworks, and abundant active sites. These characteristics significantly enhance HER kinetics, speed up electron transfer, and refine the adsorption energies of intermediates, thus boosting both catalytic activity and stability. Thus, core–shell MOF-based materials offer new strategies for developing low-cost, high-efficiency electrocatalysts for water splitting hydrogen production.

Yang proposed a hierarchical core–shell nanoarray structure and prepared RuMo/Cu_2_O@C by introducing Ru and Mo into Cu-BTC@Cu(OH)_2_ followed by pyrolysis [183]. Density functional theory (DFT) calculations revealed that the incorporation of highly dispersed Ru and Mo drives the hydrogen adsorption free energy on RuMo/Cu_2_O@C toward zero, enabling outstanding HER performance. In 1 M KOH freshwater and natural seawater, the catalyst demands overpotentials of merely 18 mV and 24 mV, respectively, to achieve a current density of 10 mA cm^−2^, significantly lower than the 41 mV observed for the commercial Pt/C electrode (Figure 10b). Moreover, the catalyst operates stably for over 800 h in alkaline seawater under an ultrahigh current density of 2 A cm^−2^ (Figure 10c). Liu et al. prepared a core–shell ZIF_800_@COF catalyst by directly pyrolyzing ZIF-67@TP-BPY-COF [187]. The uniformly dispersed Co nanoparticles together with Co-N_4_ sites provided a high density of active centers. The COF shell not only enhanced the structural stability and prevented collapse during pyrolysis but also promoted the formation of mesoporous channels and increased the degree of graphitization, thereby improving the electrical conductivity. The Tafel slope of ZIF_800_@COF is measured at 44 mV dec^−1^, notably lower than the slopes of Pt/C (93 mV dec^−1^) and ZIF_800_ (91 mV dec^−1^). This characterization highlights its outstanding bifunctional catalytic efficiency for both the ORR and the HER.

The substantial release of CO_2_ has caused serious environmental and energy challenges, whereas the transformation of CO_2_ into valuable fuels or chemicals via ECO_2_RR is viewed as a very encouraging approach for utilizing carbon resources [188,189]. However, CO_2_ is highly chemically inert, and its stable C=O bonds are difficult to break. Together with interference from the competing HER, the adsorption and activation of CO_2_ on catalyst surfaces require high energy, which imposes significant limitations on overall activity and selectivity [190]. Core–shell MOFs, through the use of designable active sites and controllable interfaces, enable efficient CO_2_ adsorption and enrichment, optimization of the adsorption energies of key intermediates, and enhanced electron and proton transport. As a result, they significantly improve the catalytic activity and product selectivity in CO_2_ reduction reactions.

Pei et al. used Mn-ZIF-8 as a precursor and employed a double-step etching and pyrolysis to prepare a yolk–shell Zn1Mn1-SNC catalyst [191]. In an H-type electrochemical cell, this material showed high CO_2_RR kinetics with a TOF of 2931.7 h^−1^ at an overpotential of 343 mV and CO Faradaic efficiency of 97%, which was much higher than that of Zn1-SNC (64%) and Mn1-SNC (39%). According to the DFT calculations, the neighboring Zn-Mn dual-atom sites co-regulate the adsorption of the key COOH* intermediate that optimizes the rate-determining step, reduces the energy barrier, and thus remarkably enhances the electrochemical reduction in CO_2_ to CO. Li et al. used HKUST-1 as a precursor and applied a directed conversion combined with a covalent functionalization strategy to successfully prepare a PDMS-functionalized core–shell PDMS-Cu_2_O/C catalyst [185]. The maximum Faradaic efficiency for ethylene production achieved by this catalyst was 71%, with a partial current density that reached 513.6 mA cm^−2^. This performance is superior to that of Cu_2_O/C (97.2 mA cm^−2^) and Cu_2_O/C@PDMS (240.9 mA cm^−2^). In situ infrared spectroscopy combined with DFT calculations indicated that altering the surface through covalent modifications adjusts the d-band center, enabling accurate regulation of the adsorption energies for CHO* and various other intermediates involved in ethylene production. This adjustment notably reduces the energy barriers for critical processes, especially the generation of CH_2_CHO* (Figure 10d). Wen and co-workers used HKUST-1 as the core to construct a core–shell HKUST-1@Cu_2_O/PTFE-1 catalyst [186]. Its high surface area and abundant active sites enhanced CO_2_ adsorption, thereby improving the electrocatalytic performance. At the same time, the hydrophobic interface formed by polytetrafluoroethylene (PTFE) effectively suppressed the HER, significantly increasing the CO_2_ reduction efficiency. This unique structure enabled extraordinary performance in hydrocarbon fuel production, achieving a C_2+_ Faradaic efficiency of 67.41% (Figure 10e). In addition, its Faradaic efficiency for ethylene is significantly higher than those of Cu_2_O/PTFE (17.98%) and HKUST-1/PTFE (10.63%). Additionally, Luo and co-workers prepared a core–shell Cu_2_O@ZIF-8 composite by growing ZIF-8 on the surface of Cu_2_O for surface modification [192]. This material enabled the complete conversion of ECO_2_RR products from C_2_H_4_ to hydrogen-rich syngas. At a relatively low potential of 0.7 V versus RHE, the H_2_:CO ratio reached 2:1, producing syngas that is one of the most important feedstocks for the alcohol production industry.

#### 3.1.3. Photocatalytic Reaction

Dependence on fossil fuels has intensified pollution and the greenhouse effect, which makes the demand for clean and renewable energy alternatives particularly urgent. Solar energy, known for its abundance and lack of emissions, is considered one of the most promising sustainable energy sources for facilitating a transition to green energy. Photocatalytic processes, which harness solar energy to transform it into chemical energy through the excitation of a catalyst by light, offer considerable promise for producing clean energy and remedying environmental issues. This includes applications such as hydrogen generation through photocatalysis, the photoreduction of CO_2_, and the degradation of pollutants [193,194]. In recent years, core–shell MOF composites have demonstrated relatively strong photocatalytic activity. In these structures, the MOF core provides a high surface area, tunable porosity, and abundant active sites, while the shell layer enhances light absorption, modulates energy levels, improves the interfacial microenvironment, and increases structural stability. The core–shell structure’s heterointerface enhances the spatial separation of photogenerated carriers which greatly enhances the photocatalytic performance of the photocatalyst [83].

The photocatalytic process of CO_2_ reduction can take place under mild conditions like room temperature and pressure while utilizing practically unlimited solar energy and water as the energy and electron sources [195]. Nonetheless, CO_2_ is stable and has a high reduction potential, implying that its photodriven conversion still requires a high energy input. At present, only limited photocatalysts are capable of efficiently utilizing solar energy to convert CO_2_. Core–shell MOFs with tunable interfacial structures, improved light-harvesting capacity and efficient charge separation could be a useful design strategy to enhance the photocatalytic CO_2_ reduction performance.

**Figure 11:**
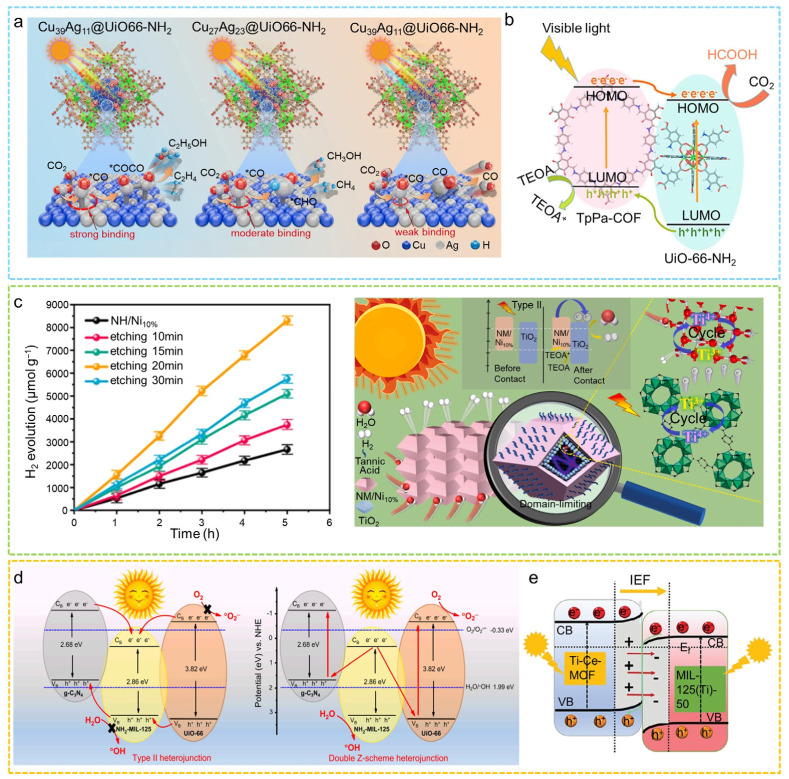
(**a**) The photocatalytic CO_2_RR regulation mechanism of Cu_x_Ag_50−x_@UiO66-NH_2_ on reduced carbon products [196]; (**b**) schematic diagram of photocatalytic reduction in CO_2_ by UiO-MOF@TpPa-COF under visible light irradiation [198]; (**c**) data on hydrogen production from various samples, along with a feasible mechanism for charge transfer and photocatalytic hydrogen generation over the TiO_2_@NM/Ni_10%_ hollow double-shell heterojunction [199]; (**d**) schematic representation of reaction mechanism of UiO-66@NH_2_-MIL-125/g-C_3_N_4_ photocatalyst [51]; (**e**) Z-scheme charge transfer mechanism of MIL-125(Ti)-50@Ti-Ce-MOF [200].

The Jiang group constructed a core–shell photocatalyst, Cu_x_Ag_50−x_@UiO-66-NH_2_, using a “dual ship bottling” strategy [196]. In this system, Cu_x_Ag_50−x_ nanoparticles are encapsulated within the pores of UiO-66-NH_2_, and the confinement effect of the MOF pores effectively prevents the aggregation of Cu_x_Ag_50−x_, greatly enhancing the photocatalytic activity. Cu_12_Ag_38_@UiO-66-NH_2_ exhibits enhanced carbon reduction rates (162.47 μmol g^−1^ h^−1^) in photocatalytic CO_2_ reduction, which is 2.83 times higher than that of UiO66-NH_2_ (57.25 μmol g^−1^ h^−1^). Mechanistic analysis reveals that when exposed to visible light irradiation, a portion of the photogenerated electrons moves from UiO66-NH_2_ to the interface of the alloy. Additionally, the varying bonding strengths between the C-Ag active sites within Cu_x_Ag_50−x_ and the *CO intermediate dictate the ensuing reaction pathways (Figure 11a). Dai and co-workers introduced the semiconductor TiO_2_ into a core–shell Cu_2_O@Cu-MOF structure to construct the composite photocatalyst Cu_2_O@Cu-MOF/TiO_2_ [197]. In this system, the separation of photogenerated charge carriers is aided by Cu_2_O(Cu^+^), whereas CO_2_ adsorption and activation are improved by the Cu-MOF(Cu^2+^). Their synergistic effects effectively lower the formation barriers of key intermediates such as *COOH and *CHO. Moreover, Cu_2_O@Cu-MOF, serving as a co-catalytic component, enables deep multi-electron reduction of *CO prior to its desorption, steering the reaction along the desired pathway and enabling selective CH_4_ formation. This photocatalyst demonstrates a substantial rate of methane production (366.0 μmol g^−1^ h^−1^), surpassing pristine TiO_2_ and Cu-MOF by factors of 25 and 724, respectively, and it also shows remarkable hydrocarbon selectivity (95.5%). Yu and colleagues developed a core–shell photocatalyst, UiO-MOF@TpPa-COF, using an epitaxial growth method [198]. This approach allows for the linkers’ free rotation to demonstrate dynamic adaptive behavior that reduces lattice mismatch. In the process of reducing CO_2_ through photocatalysis, the UiO-MOF@TpPa-COF structure facilitates a directional pathway for charge transfer, moving electrons from the shell of TpPa-COF to the core of UiO-66-NH_2_. This setup achieves almost 100% selectivity for the photochemical reduction in CO_2_ into formic acid, with an impressive rate that reaches 178.3 μmol g^−1^ h^−1^. This performance is far superior to that of UiO-66-NH_2_ (24.1 μmol g^−1^ h^−1^, 84.3%) and TpPa-COF (25.3 μmol g^−1^ h^−1^, 26.6%) (Figure 11b).

Photocatalytic hydrogen production is a sustainable technology that converts solar energy into clean hydrogen fuel, but its efficiency is often limited by insufficient light absorption, severe charge carrier recombination, and a restricted number of active sites [201]. Core–shell MOFs, with their tunable structures and hierarchical interface advantages, have shown great potential in enhancing photocatalytic hydrogen generation. In recent years, various core–shell systems have achieved significant improvements in hydrogen production rates, highlighting the promising applications of core–shell MOFs in photocatalytic water splitting. Wang et al. prepared a sandwich-structured UiO-66-NH_2_@Pt@UiO-66-X (X = -H, -Br, -Cl, etc.) via epitaxial growth. This design facilitates close interaction among the UiO-66-X shell, the UiO-66-NH_2_ core, and the Pt nanoparticles [202]. Simultaneously, it enables the -X functional groups on the shell’s MOF to generate a customized microenvironment that accurately regulates the interactions between the UiO-66-NH_2_ core and the Pt NPs. In a photocatalytic HER using triethylamine as the electron donor, UiO-66-NH_2_@Pt@UiO-66-H exhibited the highest activity, reaching 2708.2 μmol g^−1^ h^−1^. Huec et al. synthesized a heterostructured MOF@MOF composite (UiO-66-NH_2_@MIL-88B), which achieved an apparent quantum yield of approximately 0.9% for photocatalytic overall water splitting at 400 and 450 nm [203]. Within 3 h, the UiO-66-NH_2_@MIL-88B exhibited hydrogen and oxygen evolution activities of 690 and 279 μmol g^−1^, respectively. Moreover, to tackle the swift recombination of photogenerated electron-hole pairs in photocatalytic hydrogen production using MOFs, Li et al. suggested a method of forming heterostructures by combining MOFs with various semiconductor materials to improve the photocatalytic efficiency [199]. The researchers utilized tannic acid as a protective agent to in situ fabricate a hollow double-shell heterostructure, TiO_2_@NM/Ni_10%_. Following 20 min of etching, the TiO_2_@NM/Ni_10%_ structure demonstrated efficient hydrogen evolution at a rate of 1659.9 μmol h^−1^ g^−1^, which is roughly 2.37 times greater than the performance of the unaltered NM/Ni_10%_ at 699 μmol h^−1^ g^−1^. This improvement can primarily be linked to the dual-cycle transfer of electrons between Ti^4+^ and Ti^3+^, the swift separation of charges facilitated by the type-II heterojunction, and the increased light absorption due to the confinement effect provided by the hollow double-shelled structure. This study presents a novel approach for designing high-performance core–shell MOF photocatalysts. (Figure 11c).

Photocatalysis has gained considerable interest as an eco-friendly, sustainable, and economical treatment technology, as it is capable of utilizing solar energy to thoroughly break down organic contaminants into non-toxic byproducts like CO_2_ and H_2_O. During the photocatalytic degradation process, the photogenerated electron-hole pairs in the catalyst can directly participate in redox reactions with pollutants or produce highly reactive radicals, enabling deep decomposition of organic contaminants. Therefore, improving the activity, stability, and recyclability of photocatalysts has become a critical focus in current research on environmental remediation [204]. Sepehrmansourie et al. grew the core–shell UiO-66-on-MIL-125 on g-C_3_N_4_ nanosheets to construct a double Z-scheme heterojunction, UiO-66@NH_2_-MIL-125/g-C_3_N_4_ [51]. When exposed to light, this photocatalyst facilitated the degradation of the antibiotic ofloxacin (OFL) at a rate roughly 1.79 times greater than that of unmodified UiO-66@NH_2_-MIL-125, with visible light enhancing the effective separation of photogenerated electron-hole pairs. Reactive species such as ·OH and ·O^2−^, were instrumental in the photodegradation process of OFL. (Figure 11d). Wang et al. constructed a core–shell hybrid material, NH_2_-UiO-66@DAT-HOF (U@H), via molecular-level covalent bonding [205]. The DAT-HOF shell uniformly coated the MOF core, extending the photocatalyst’s visible light response and effectively promoting photogenerated electron-hole separation through an S-scheme heterojunction. The synergistic interaction between the core and shell significantly enhanced both the structural stability and photocatalytic activity, resulting in a TC degradation efficiency approximately 60.7 times higher than that of the parent material. Similarly, Hu et al. constructed a yolk–shell heterostructure, MIL-125(Ti)@Ti-Ce-MOF, through a combined ion-etching and reconstruction strategy [200]. The controllable built-in electric field within this architecture effectively separated photogenerated electrons and holes, enabling spatially directed charge transport and markedly improving carrier separation and migration efficiency. Benefiting from these synergistic effects, the photocatalytic degradation performance toward TC was enhanced by approximately 52.5-fold compared with Ti-Ce-MOF and by 5.5-fold relative to MIL-125(Ti) (Figure 11e).

### 3.2. Gas Adsorption Separation

Hierarchically structured core–shell MOF composites have demonstrated remarkable advantages in gas separation and purification. By incorporating functional gradients, tunable pore apertures, and interfacial synergistic effects within a single material, core–shell architectures enable efficient molecular sieving and selective adsorption of gas species [92]. For any gas separation application, an ideal adsorbent should simultaneously exhibit high capacity, high selectivity, and unparalleled structural stability requirements that core–shell MOFs are well positioned to meet. In recent years, extensive research has focused on regulating core–shell interfaces, channel architectures, and chemical microenvironments to achieve the precise recognition and efficient capture of various gas molecules, including CO_2_, CH_4_, C_2_H_2_, C_2_H_4_, and VOCs. This section provides a systematic discussion of recent advances in core–shell MOF materials for several representative gas adsorption separation systems, such as CO_2_ capture from flue gas [206,207], natural gas purification [138,208], and acetylene refinement [45,209]. The aim is to clarify the relationships between structure and properties, providing valuable insights for the development of efficient materials for gas separation.

#### 3.2.1. CO_2_ Capture from Flue Gas

Post-combustion CO_2_ capture is a crucial technology option for reaching a global energy transition towards low-carbon. The low CO_2_ concentration and complicated composition of flue gas impose dual challenges of selectivity and stability on the adsorbents. Typical flue gas is considered as N_2_ at around 70–75%, CO_2_ at 15–16% and H_2_O at 5–7%. Therefore, it is necessary to develop advanced materials having high CO_2_ affinity yet moisture-resistant under multicomponent competitive condition [138]. The increasing advantages of core–shell MOFs have emerged as a recent phenomenon for flue gas CO_2_ capture because of their hierarchical architectures. The shell can effectively screen N_2_, while the core provides a high-capacity CO_2_ adsorption site, which synergistically achieves the high selectivity and fast transport of CO_2_. The literature has summarized representative core–shell MOF materials suitable for CO_2_/N_2_ separation in order to systematically compare their adsorption behaviors (Table 2). The efficient capture of CO_2_ requires materials with a high affinity for this gas, high selectivity and stable interface. Composite materials made of core–shell MOFs provide an exciting new option for CO_2_ capture from flue gas.

In 2013, Li and co-workers proposed a strategy to enhance CO_2_ capture from flue gas by constructing core–shell MOF composites [210]. In their design, the core consisted of a porous mixture of bio-MOF-11/14, while the shell was composed of bio-MOF-14, which has a lower porosity but superior water resistance. The core–shell MOF produced displayed a 30% increase in CO_2_ adsorption capacity compared to the pure bio-MOF-14 and had a reduced N_2_ uptake in relation to the core MOF. Additionally, water stability tests indicated that the moisture-resistant bio-MOF-14 shell successfully shielded the core, which is sensitive to humidity, from degradation in humid environments. In 2016, Sorribas and colleagues fabricated core–shell microspheres approximately 4 mm in diameter by growing NH_2_-MIL-53(Al) on the surface of silica, yielding Silica-NH_2_-MIL-53(Al) core–shell beads [211]. The “breathing effect” of NH_2_-MIL-53(Al) effectively regulates the diffusion pathways through which CO_2_ and other molecules access the mesoporous silica core. At a CO_2_ partial pressure of 3.5 MPa, the Silica-NH_2_-MIL-53(Al) core–shell microspheres exhibited a CO_2_ uptake of approximately 10 mmol g^−1^. To enhance the understanding of how CO_2_ and N_2_ are adsorbed at the molecular level in core–shell MOFs, Klomkliang and colleagues explored the capture and separation of CO_2_ in nitrogen environments by integrating Grand Canonical Monte Carlo (GCMC) simulations with experimental studies [219]. Initially, they produced core–shell zeolitic imidazolate frameworks (ZIFs), specifically ZIF-8@ZIF-67 and ZIF-67@ZIF-8, through a seed-mediated growth technique. Among these materials, ZIF-67@ZIF-8 demonstrated a 38% enhancement in its capacity to adsorb CO_2_ and a 25% increase in selectivity for CO_2_ over N_2_ when compared to the unmodified ZIF-8. The GCMC simulations further revealed that the Zn-Co interface in the core–shell MOFs enhances CO_2_ binding energy while simultaneously reducing their affinity for N_2_.

**Figure 12:**
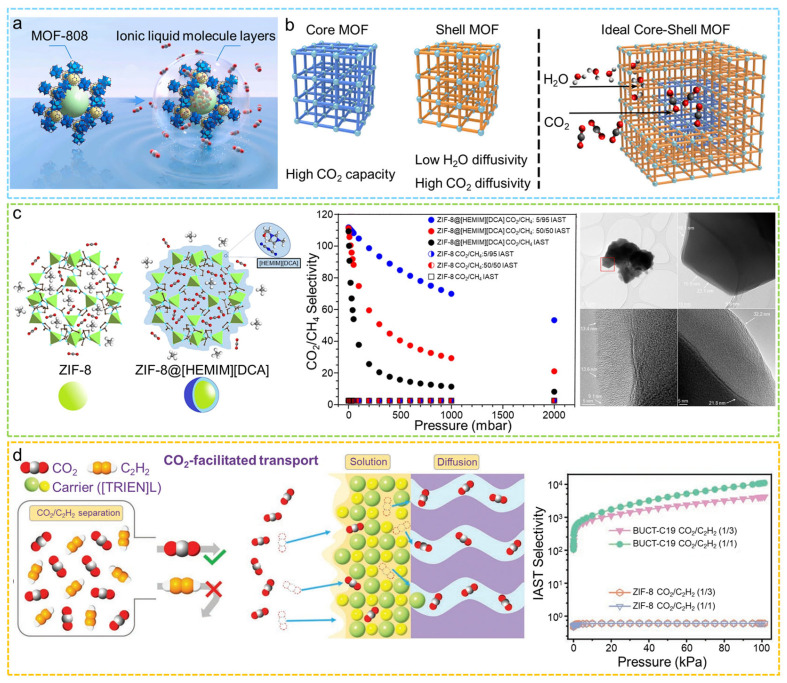
(**a**) Solution-mediated assembly of ILMLs on MOF-808 [212]; (**b**) design strategy of optimal core–shell MOF for CO_2_ capture [220]; (**c**) synthesis concept of core–shell MOF@IL structure, the ideal adsorption selectivity and IAST selectivity of ZIF-8 and ZIF-8@[HEMIM][DCA] composites at 298 K (the CO_2_/CH_4_ selectivity of ZIF-8 is relatively low, resulting in overlapping curves.), and the TEM images of ZIF-8@[HEMIM][DCA] composites (the TEM image in the upper right corner is an enlarged view of the area marked by the red box) [221]; (**d**) diagram illustrating the separation process of BUCT-C19, along with the IAST selectivity for gas mixtures at various ratios involving both BUCT-C19 and ZIF-8 at 298 K [45].

Furthermore, Zhao and colleagues constructed a core–shell MOF by assembling an ionic liquid molecular layer (ILML) to MOF-808 [212]. By adjusting the IL layer’s thickness, they were able to find a balance between the capacity for CO_2_ adsorption and the selectivity (Figure 12a). At ambient temperatures, the material exhibited a CO_2_ uptake of 3.00 mmol g^−1^, which is 2.6 times higher than that of pristine MOF-808. Under simulated industrial flue gas conditions, the CO_2_/N_2_ selectivity reached as high as 372 (CO_2_/N_2_ = 15/85). He et al. designed a core–shell composite by computationally selecting the core and shell components to achieve distinct functions [220]. In this design, the core MOF acts as the main adsorbent selective for CO_2_, whereas the shell acts as a barrier to impede the diffusion of H_2_O into the core (Figure 12b). The research revealed that incorporating a shell that exhibits high selectivity for CO_2_ over H_2_O diffusion can considerably reduce the negative impact of moisture on CO_2_ adsorption.

#### 3.2.2. Natural Gas Purification

As an alternative to coal and crude oil, natural gas has attracted widespread attention. Its main component is methane (CH_4_, 30–98%), along with impurities such as CO_2_, N_2_, and H_2_S. The presence of these impurities not only reduces the calorific value of natural gas but also poses risks of corrosion to equipment and pipelines [133,222]. Therefore, the effective separation of these contaminant gases is both technically and economically important for improving natural gas quality. In recent years, core–shell MOF composites have shown significant potential for removing impurities such as CO_2_ and N_2_ from natural gas.

In 2018, Zeeshan and co-workers introduced a new concept of core–shell MOF@IL composites [221]. They constructed a core–shell ZIF-8@[HEMIM][DCA] composite by depositing 1-(2-hydroxyethyl)-3-methylimidazolium dicyanamide ([HEMIM][DCA]) onto the surface of ZIF-8. High-resolution TEM combined with XPS depth-profiling confirmed that [HEMIM][DCA] was deposited on the outer surface of ZIF-8. The ionic liquid layer on the ZIF-8 surface functions as a smart gate, exploiting the large difference in solubility of CO_2_ and CH_4_ in the IL to achieve a markedly enhanced CO_2_ adsorption capacity (approximately 5.7 times that of pristine ZIF-8) and CO_2_/CH_4_ selectivity (approximately 45 times that of pristine ZIF-8). Unlike conventional IL-incorporated MOFs, the core–shell structure preserves the accessibility of the MOF pores, significantly improving the gas separation performance (Figure 12c). Similarly, Guo et al. combined the CO_2_-philic amino-functionalized ionic liquid triethylenetetramine lysinate ([TETA][Lys]) with ZIF-8 via a dissolution–diffusion mechanism to construct a core–shell ZIF-8@[TETA][Lys] solid adsorbent [213]. Owing to the significantly higher CO_2_ solubility in the outer IL layer, the material can selectively capture CO_2_ while excluding other gas molecules, exhibiting extremely high IAST selectivities for CO_2_/CH_4_ (2.4 × 10^6^, 15/85, *v*/*v*), CO_2_/N_2_ (1 × 10^8^, 10/90, *v*/*v*), and CO_2_/C_2_H_2_ (1899, 50/50, *v*/*v*) separations. Moreover, the material also demonstrated effective separation performance under simulated flue gas treatment and natural gas purification conditions. Liu and colleagues synthesized ZIF-8 atop the ZIF-L(Co) surface to create a core–shell structure known as ZIF-L(Co)@ZIF-8 [223]. This composite was subsequently integrated into Pebax to produce mixed matrix membranes (MMMs) that exhibited improved performance in CO_2_/CH_4_ separation. The ZIF-8 outer layer contributed to an increased affinity for CO_2_, whereas the inner ZIF-L(Co) facilitated the molecular sieving of CO_2_ and CH_4_. Furthermore, the sheet-like configuration of ZIF-L(Co)@ZIF-8 established a complex pathway within the membrane, which further hindered the transport of CH_4_ and notably enhanced both the gas separation efficiency and mechanical properties. Of the membranes tested, ZIF-L(Co)@ZIF-8/Pebax-3 exhibited the highest performance with a CO_2_ permeability of 145.7 Barrer and CO_2_/CH_4_ selectivity of 40.9, corresponding to increases of 79.1% and 110.1% compared to the pristine Pebax membrane. Moreover, it is important to remove H_2_S from natural gas as it is corrosive to pipelines and will produce toxic sulfur oxides when combusted that contribute to acid rain. To this end, Fakhraie et al. constructed a UiO-66(Zr) shell on porous MIL-101(Cr), obtaining a core–shell MIL-101(Cr)@UiO-66(Zr) (MU) structure by adjusting the shell growth time [214]. The results showed that MU exhibited adsorption capacities for H_2_S and CO_2_ that were 84.4% and 121.8% higher than those of pristine UiO-66(Zr), respectively. Moreover, its selectivities for H_2_S/CH_4_, H_2_S/N_2_, CO_2_/CH_4_, and CO_2_/N_2_ were significantly enhanced compared with MIL-101(Cr), increasing by 7.8%, 47.0%, 32.1%, and 59.9%, respectively. This high-efficiency separation is attributed not only to the newly introduced open metal sites but also to the size-sieving effect resulting from the optimized pore sizes of the MU nanocrystals.

#### 3.2.3. Acetylene Purification

Acetylene (C_2_H_2_), as a high-value chemical feedstock, has a purity that directly impacts the efficiency and safety of downstream fine chemical and catalytic processes. However, industrial acetylene often contains impurities such as CO_2_ and C_2_H_4_ [224]. These gases are very similar to C_2_H_2_ in terms of molecular size, polarity, and adsorption behavior, making conventional distillation and solvent absorption processes energy-intensive and limited in selectivity. The recent development of core–shell MOFs has provided a new avenue for acetylene separation and purification. Applying a shell of tunable pore dimensions and surface chemical properties on a high-porosity–high-adsorption-capacity MOF core allows us to achieve accurate molecular sieving with a high capacity of the core material. The beneficial effect of the shell on the overall selectivity is thus attributed to its size exclusion and interface modulation effects towards impurities like CO_2_ and C_2_H_4_, which promote the preferential transport and adsorption of C_2_H_2_.

The Liu group developed a core–shell MOF@IL material (BUCT-C19) using an impregnation method with ZIF-8 as the core and deposited triethylenetetramine lactate by layering it on the surface [45]. Due to the good solubility of CO_2_ in the ionic liquid and the near-complete exclusion of C_2_H_2_, the resulting material promoted molecular sieve-based CO_2_/C_2_H_2_ separation, achieving dominant adsorption of CO_2_ over C_2_H_2_. The IAST selectivity for a 1:1 CO_2_/C_2_H_2_ mixture reached as high as 10^4^ (Figure 12d). The actual separation performance of BUCT-C19 for CO_2_/C_2_H_2_ gas mixtures was evaluated through breakthrough experiments, demonstrating its ability to produce high-purity C_2_H_2_ gas (≥99.9%). This solution-based separation strategy using MOF@IL (BUCT-C19) effectively avoids the need for precise structural tuning of the adsorbent. Liang and colleagues employed a mechanochemical “Cage-on-MOF” strategy, using porous coordination cages (PCCs) as surface modifiers, successfully constructing 28 different MOF@PCC composites [209]. By tuning the combinations of MOFs and PCCs, the C_2_H_2_ adsorption capacity of MOF-808@PCC-4, with highly matched window sizes, was significantly enhanced (+64%), and the breakthrough time for CO_2_/C_2_H_2_ separation was extended by approximately 40%. Meanwhile, MIL-101@PCC-4 achieved a C_2_H_2_ adsorption capacity as high as 6.11 mmol g^−1^. Notably, this strategy exhibits excellent scalability, enabling the production of 100 g MOF@PCC composites in just 5 min.

## 4. Conclusions and Prospects

As functional material design continues to advance, core–shell MOF composites have received considerable attention for their unique structural advantages. This paper systematically summarizes the key structural features, major classification schemes, typical synthetic strategies, and recent progress of core–shell MOF composites in important fields such as catalysis and gas adsorption and separation. Core–shell MOFs, a highly programmable class of multifunctional materials, enable the synergistic integration of different components through a carefully designed core shell architecture. These materials can overcome the weaknesses of a single MOF in terms of stability, selectivity, and functional diversity due to the unique physicochemical synergistic effect between the two at their interface. As a result, they possess considerable scientific and application value. The construction models of core–shell structures, namely MOF@MOF, MOF@COF, MOF@oxide/carbon materials, MOF@ionic liquid/polymer, and A@MOF architectures, can effectively improve their structural tunability and functional integration.

From the angle of material system construction, structural diversity is one of the most striking features of core shell MOFs. Core–shell compositions are not simple combinations of a “core” and a “shell” type. Rather, they take advantage of deeper synergistic effects, manifesting themselves in their structural gradients, functional partitioning and interfacial chemical coupling. For instance, many MOF@COFs have a rigid–flexible heterointerface that maintains the MOF’s adsorption capability and enhances stability and selectivity. MOF@carbon or MOF@oxide systems utilize carbon layers or inorganic shells to enhance their electrical conductivity, thermal stability, or moisture resistance. These diverse core–shell MOF architectures will enrich the structural variety of the materials and thus provide a powerful platform for fine-tuning their application performance.

In the synthetic strategies of core–shell MOFs, diversification and precision show concurrent advancing trends. Epitaxial growth is based on a lattice matching phenomenon and can be used in the oriented construction of shell layers. However, this method has limited applications and generally is not suitable for the design of heterogeneous structures. Methods that entail the use of seeds on the core surface to create nucleation sites are capable of extending the material scope and improving shell continuity. In addition, more rapid and efficient one-pot approaches are used that allow for the quick and simultaneous formation of the core and shell of these materials, which thereby makes them appealing for production at scale. At the same time, post-synthetic modification strategies offer larger flexibility for shell functionalization, interfacial adjustment of chemicals, and the addition of heterogeneous components. The ongoing developments and coalesced use of such synthetic strategies allow a high precision control over shell thickness, uniformity, pore structure, and interfacial coupling. These accomplishments provide a robust foundation for bolstering the stability, selectivity, and multifunctionality of core–shell MOFs under intricate conditions.

The behaviors of the species in the core–shell structures are discovered to exhibit a strong synergism in functional performance. A stable shell, such as a COF or a carbon layer or polymer, can significantly enhance the stability of a MOF under humid, acidic or basic, and thermal stress conditions, removing major barriers to practical applications. Core–shell architectures with heterogeneous interfaces in catalysis enhance the electron transfer, charge carrier separation, reactant diffusion pathways, and integration of multiple active sites, and simultaneously improve the activity, selectivity, and stability in the reactions including CO_2_ conversion, water splitting, and fine chemical synthesis. In gas separation, a core–shell structure composed of a high-capacity core and a shell with molecular sieve separation performance is highly advantageous. By using “intelligent gating”, the selectivity can be enhanced, such as flue gas CO_2_ capture, natural gas and acetylene purification.

Although core–shell MOFs have made great strides, there are still many challenges and opportunities before practice. Future research pertaining to core–shell MOF composites should focus on the following aspects and opportunities:

(1) At present, the formation of core–shell structures is observed mostly based on some trial conditions and limited correlation; it is not systematic involving interfacial thermodynamics, nucleation kinetics, and pathways. The future research should employ in situ characterization methods (for example in situ TEM, XRD analysis, Atomic Force Microscope (AFM), and time-resolved spectroscopy) combined with multiscale computations to better understand shell growth mechanisms, allowing us to move from being “controllable” to “predictable” in our construction.
(2) Core–shell MOFs have limited practical applications due to the high solvent consumption, energy-intensive reactions, and prolonged preparation times. Future studies ought to investigate aqueous-phase syntheses, recyclable solvent systems, room temperature or low-temperature reaction pathways, and continuous-flow reactors to decrease material costs considerably and enable pilot-plant production and industrialization.
(3) Under the “dual-carbon” strategy, core–shell MOFs may hold great promise to enable direct air capture (DAC), low-temperature CO_2_ catalytic conversion, and moist flue gas separation. In separation engineering, greater attention should be paid to core–shell MOF evaluation in membrane separation, in adsorption columns and in pilot-scale reactors for moving from material-level performance to engineering-level performance. In the energy field, core–shell MOF-derived structures can serve as electrodes, energy storage materials, or supports for single-atom catalysts to further improve interfacial stability and cycling durability.
(4) Using machine learning to construct performance prediction models to develop core–shell structures as well as high-throughput computation and automated experimental platforms can help discover and rapidly screen new core–shell MOFs. Simultaneously, multiscale simulations can help elucidate the interfacial electronic structures, diffusion behaviors, and catalytic mechanisms, which could serve as the computational guidance to rationally design advanced materials.

The core–shell MOF materials show the combination of rapid development and wide application prospects. Through a better understanding of the structural formation mechanism, advances in interfacial engineering strategies, breakthroughs in structural design, and the acceleration of integration into engineering applications, core–shell MOFs are expected to make an indispensable contribution to the key areas related to sustainable development, like energy conversion, environmental remediation, and intelligent manufacturing.

## Figures and Tables

**Table 1:** Pore structures, core/shell dimensions and applications of MOF-based core–shell composites.

Composite Type	Core Material	Shell Material	MOF Surface Area (m^2^/g)	MOF Pore Size (nm)	Core–Shell MOF Surface Area (m^2^/g)	Core–Shell MOF Pore Size (nm)	Core Size (nm)	Shell Thickness (nm)	Application	Ref.
MOF@MOF	NH_2_-MIL-125	UiO-66	813	1.85	774	1.85	200–250	230–300	Photocatalytic degradation	[51]
	PCN-134	PCN-222	263.53	3.25	610.66	5.09	3000	-	Photocatalytic degradation	[68]
	ZIF-8	ZIF-67	1323.62	1.11	1402.15	1.2	-	-	Hydrogen storage	[76]
	ZIF-67	ZIF-8	1392.30	1.01	1271.82	1.02	-	-		
	MOF-808_seed_	MOF-808	669	-	1081	-	-	-	-	[73]
	NH_2_-MIL-125	ZIF-67	1298	1.90	1575	1.45	100–400	-	Adsorption	[77]
	UiO-66-N	ZIF-67	1208	-	1333	-	-	-	Hydrogenation reaction	[89]
	UiO-67	Zr-AzoBDC	~2224	-	1106	-	80.7	16.7	Light-emitter material	[90]
	UiO-67	Zr-StilBDC	~2224	-	710	-	76.6	5.21		
	UiO-66-NH_2_	ZIF-8	792.45	1.04	1563.9	1.43	-	-	Adsorption	[91]
MOF@COF	NH_2_-UiO-66	TFPT-DETH	1704	0.8	1051	0.42, 0.8	150–200	~20	Photocatalytic hydrogen	[54]
	UiO-66-NH_2_	TAPB-DMTP-COF	1298	-	1327	-	-	20	Electrochemical detection	[55]
	UiO-66-NH_2_	TapbTp	1431	-	923	-	-	-	Drug testing	[56]
	M68N	In-TCPP	675.9	-	798.4	-	-	-	Photocatalytic reduction	[83]
	ZIF-67	COF-TFP-DMPA	1382	-	510	-	4000	100	Sensing	[104]
	UiO-66-NH_2_	TpPa-1	887	-	723	-	~300	~45	Gas adsorption	[115]
	Pd/UiO-66-NH_2_	COP-1	686	1.5	241	1.25	-	-	Hydrogenation reaction	[116]
	UiO-66-(COOH)_2_	TpPa	453	~0.5	496	0.5, 1.69	-	106	D-K tandem reaction	[117]
	Ti-MOF	TpTt-COF	1385.1	-	1137.4	-	-	-	Photocatalytic cascade reactions	[107]
MOF@oxide materials	MIL-125-NH_2_	TiO_2_	1169	-	780	-	-	-	Photocatalytic H_2_ production	[121]
	MIL-101	mSiO_2_-YS	2822	1.2–2.8	1139	4.0–13.8	-	~25	Catalytic cycloaddition	[123]
		mSiO_2_-CS			1287	2.8–4.0	-	~25		
	ZIF-8	mSiO_2_	-	-	-	-	90–95	40–45	Adsorption	[124]
MOF@carbon materials	MIL-101(Cr)/S	GNS	399	-	100	-	-	-	Li-S batteries	[125]
	ZIF-8-C	PZS	927	-	929	-	60	10	Capacitive deionization	[126]
	IRMOF-1	amorphous carbon	3450	-	3130	-	-	-	Hydrophobic material	[130]
MOF@ionic liquid/polymer	ZIF-67	PDA	1298.21	0.75	804.1	0.89	-	-	Gas adsorption	[61]
	HKUST-1	PIN	798.7	-	124.8	-	-	-	Solid electrolyte	[60]
	MOF-808(Zr)	IHG	1700	1.6	1537	1.6	-	~15	Gas adsorption	[137]
	ZIF-8	PIL-Br	1294	-	1011	-	-	~20	Catalytic cycloaddition	[141]
	ZIF-8	[TETA]L	1341.0	-	473.5	-	-	~20	Gas adsorption	[138]
	MIL-101(Cr)	PANI	2681.8	2.3	1186.0	3.4	-	-	Adsorption	[142]
	MOF-801	MPD-PDTC	773	-	607	-	-	2–8	Adsorption	[145]
	MOF-801	TAM-6FDA	258	-	224	-	260	8	Oxidation reactions	[146]
A@MOF	FeTPPs	Cu-BTC	1593.8 ^1^	0.5, 0.9 ^2^	1541.2	0.5, 0.9	-	-	Gas adsorption	[148]
	CN	ZIF-8	192 ^1^	0.8 ^2^	211	0.8	-	-	Hydrophobic material	[149]
	Fe_2_O_3_	Ni-MOF-74	1207 ^1^	2.3, 2.5, 2.6 ^2^	1117	2.4, 2.5, 2.7, 3.5	-	-	Gas adsorption	[150]
	5A	ZIF-8	880 ^1^	-	379	-	-	-	Gas adsorption	[152]

^1^ The specific surface area of the shell MOF in A@MOF; ^2^ the pore size of the shell MOF in A@MOF.

**Table 2:** Core–shell MOF composites for the CO_2_/N_2_ separation.

Core–Shell MOF	CO_2_ Uptake (mmol g^−1^)	T (K)	P (bar)	S_CO2/N2_ ^1^	Ref.
CSS-50	3.4	298	1	-	[41]
MOF@IHG	2.51	298	1	1780.6 ^2^	[137]
IL-ZIF-IL	1.53	298	1	5570 ^2^	[138]
M@iCOP-50	3.33	273	1	75 ^5^	[162]
M@COF-0.10	4.04	273	1	153 ^3^	[163]
ZIF-11@ZIF-8	8.21	298	3.45	40.2 ^5^	[208]
II@bio-MOF-14	2.60	273	1	-	[210]
MSS-NH_2_-MIL-53(Al)	10.0	273	35	-	[211]
ILML	3.0	298	1	372 ^2^	[212]
ZIF-8@[TETA][Lys]	2.51	298	1	108 ^3^	[213]
MU-8	2.41	298	1	7.4 ^5^	[214]
MIL-101(Cr)@TP	1.96	298	1	10,943 ^4^	[215]
Mg-MOF-74@ZIF-8-3	3.56	298	1	232 ^2^	[216]
MOF-CNFs	2.71	298	1	66.0 ^2^	[217]
NH_2_-UiO-66@Br-COF-4	3.85	273	1	58.9 ^5^	[218]

^1^ The S_CO2/N2_ was calculated by (ideal adsorbed solution theory) IAST selectivity; ^2^ CO_2_/N_2_ = 15/85 (*v*/*v*); ^3^ CO_2_/N_2_ = 10/90 (*v*/*v*); ^4^ CO_2_/N_2_ = 50/50 (*v*/*v*); ^5^ CO_2_/N_2_ ratio was not indicated.

## Data Availability

No new data were created or analyzed in this study.
